# NASICON-Structured NaTi_2_(PO_4_)_3_ for Sustainable Energy Storage

**DOI:** 10.1007/s40820-019-0273-1

**Published:** 2019-05-25

**Authors:** Mingguang Wu, Wei Ni, Jin Hu, Jianmin Ma

**Affiliations:** 1 School of Physics and Electronics, Hunan University, Changsha, 410082 People’s Republic of China; 2 Faculty of Technology, University of Oulu, 90014 Oulu, Finland; 3 Panzhihua University, Panzhihua, 617000 People’s Republic of China; 4 Key Laboratory of Materials Processing and Mold, Ministry of Education, Zhengzhou University, Zhengzhou, 450002 People’s Republic of China

**Keywords:** NaTi_2_(PO_4_)_3_, Sodium superionic conductor, Anode, Batteries, Hybrid capacitors

## Abstract

Several emerging energy storage technologies and systems have been demonstrated that feature low cost, high rate capability, and durability for potential use in large-scale grid and high-power applications. Owing to its outstanding ion conductivity, ultrafast Na-ion insertion kinetics, excellent structural stability, and large theoretical capacity, the sodium superionic conductor (NASICON)-structured insertion material NaTi_2_(PO_4_)_3_ (NTP) has attracted considerable attention as the optimal electrode material for sodium-ion batteries (SIBs) and Na-ion hybrid capacitors (NHCs). On the basis of recent studies, NaTi_2_(PO_4_)_3_ has raised the rate capabilities, cycling stability, and mass loading of rechargeable SIBs and NHCs to commercially acceptable levels. In this comprehensive review, starting with the structures and electrochemical properties of NTP, we present recent progress in the application of NTP to SIBs, including non-aqueous batteries, aqueous batteries, aqueous batteries with desalination, and sodium-ion hybrid capacitors. After a thorough discussion of the unique NASICON structure of NTP, various strategies for improving the performance of NTP electrode have been presented and summarized in detail. Further, the major challenges and perspectives regarding the prospects for the use of NTP-based electrodes in energy storage systems have also been summarized to offer a guideline for further improving the performance of NTP-based electrodes.

**Electronic supplementary material:**

The online version of this article (10.1007/s40820-019-0273-1) contains supplementary material, which is available to authorized users.

## Highlights

- For the first time, we fully presented the recent progress of the application of NaTi_2_(PO_4_)_3_ on sodium-ion batteries including non-aqueous batteries, aqueous batteries, aqueous batteries with desalination, and sodium-ion hybrid capacitors.
- The unique NASICON structure of NaTi_2_(PO_4_)_3_ and the various strategies on improving the performance of NaTi_2_(PO_4_)_3_ electrode have been presented and summarized in detail.

**Electronic supplementary material:**

The online version of this article (10.1007/s40820-019-0273-1) contains supplementary material, which is available to authorized users.

## Graphical abstract

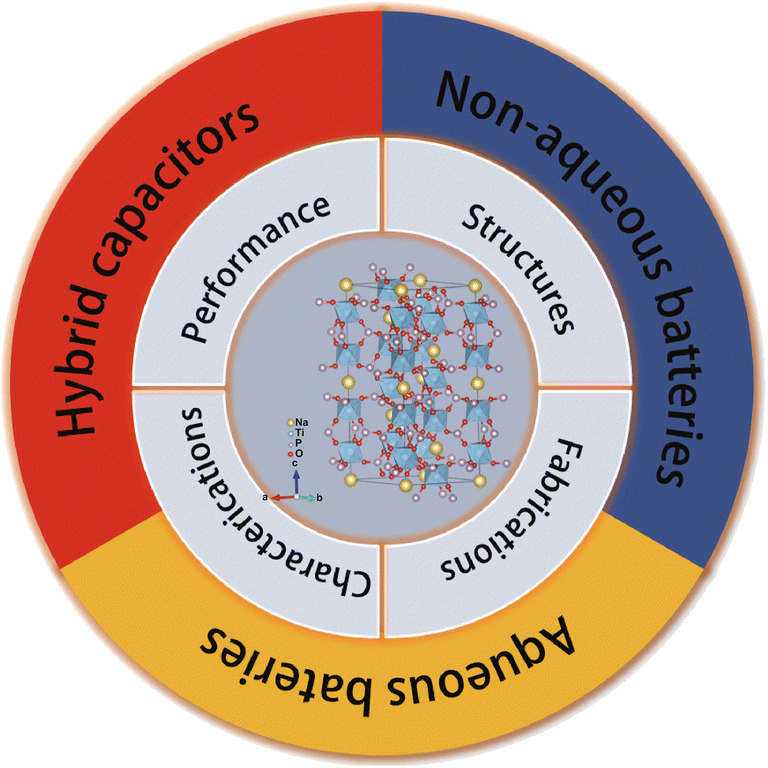

## Introduction

In recent years, with the increasing consumption of fossil fuels, numerous studies have investigated the development of various types of renewable and clean energy devices [1–10]. Among current technologies, lithium-ion batteries (LIBs) have been considerably developed and widely used in portable electronic devices and large-scale grid storage applications because of their high energy density and long lifespan [11, 12]. However, the limited lithium resources and the rising cost of LIBs have stimulated research on the similar sodium-ion batteries (SIBs) on account of sodium’s abundance in nature and environmental benignity [13–20]. At the same time, some other sodium-based energy devices such as aqueous batteries with desalination have also been developed due to their wide range of applications [21, 22].

Generally, both LIBs and SIBs rely on the reversible intercalation and deintercalation process of lithium or sodium ions between the positive and negative electrodes via the electrolyte during the charging and discharging process to complete energy conversion [13]. In particular, active electrode materials, especially anode materials, play an important role in the performance of batteries. In that regard, the development of suitable anode materials with high capacity, long cycle life, and excellent rate performance is of significant importance [15, 18, 23–28].

At present, although research on LIBs and SIBs has made considerable progress, and the most advanced batteries usually have high energy density, the slow kinetics of ion intercalation and deintercalation limits the achievement of higher power density and better rate performance [14, 25, 29].

Supercapacitors (SCs), characterized by electric double-layer capacitors (EDLCs), are a new type of energy storage device that stores energy by physical adsorption/desorption of an electric charge to form an electric double layer on the electrode-electrolyte interface. The excellent electrode dynamics of physical absorption and desorption enable features such as high power capability, excellent cycling performance, and long lifespan [30, 31]. However, EDLCs are inferior to batteries in terms of energy density [32, 33]. Therefore, merging the merits of the high-energy-density battery with the Faradaic electrode and high-power-density supercapacitor with the non-Faradaic electrode to develop a hybrid capacitor is a promising strategy to increase the energy density without sacrificing the high power density and long lifetime [30, 34–37].

**Fig. 1:**
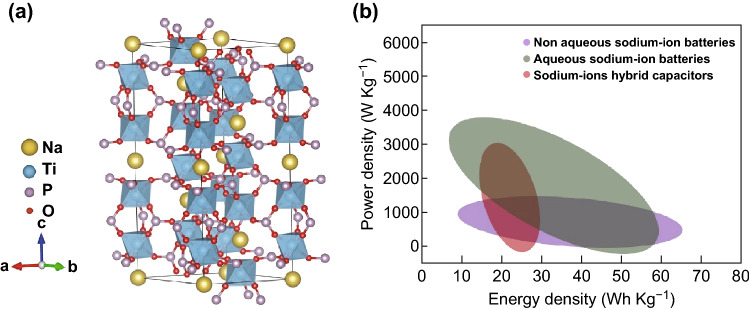
**a** Crystal structure of NaTi_2_(PO_4_)_3_ and **b** Ragone plot of NaTi_2_(PO_4_)_3_-based materials for non-aqueous sodium-ion batteries, aqueous sodium-ion batteries, and sodium-ion hybrid capacitors [32, 43, 44, 46, 95, 127, 131, 132, 159, 164, 165, 218]

Recently, NASICON (sodium superionic conductor) has gained tremendous attention as a promising anode material due to both its outstanding ion conductivity and high-voltage platform. The typical chemical formula of NASICON is A_*x*_MM′(XO_4_)_3_, where A, M & M′, and X represent metallic elements (e.g., Li, Na, and K), transition metal elements (e.g., Ti, V, and Mn), and nonmetallic elements (e.g., P and S), respectively [25, 38–41]. Among these compounds, NaTi_2_(PO_4_)_3_ (NTP) with an open framework is considered to be the optimal electrode material for SIBs due to its 3D open framework, in which TiO_6_ octahedra share all corners with PO_4_ tetrahedra, thereby providing a large interstitial space for Na^+^ ion diffusion as shown in Fig. 1a [25, 32]. In recent years, many studies on the application of NTP in SIBs, including non-aqueous batteries, aqueous batteries, aqueous batteries with desalination, and sodium-ion hybrid capacitors, have been reported (see the typical examples in Tables S1 and S2) [42]. For example, one limitation of the present electrochemical system that is observed in full cells is capacity fade at low rates less than 1 C. By demonstrating high rate capability upon removal of the electronic conductivity limitation, the state-of-the-art results show that NTP has sufficiently high sodium chemical diffusivity to provide 100 C rate capability at submicron crystallite sizes, i.e., up to 70% of the NTP capacity can be obtained with a discharge time of 36 s. The aqueous Na-ion system, e.g., NTP/NMO, is capable of exceptionally high charge/discharge rates (over 100 C) and stable cycling to > 1000 cycles, while delivering severalfold higher energy densities than supercapacitors [43]. Moreover, some new Na-ion hybrid capacitor (NHC) systems with organic electrolytes could deliver a high energy density of, e.g., ≈ 80 Wh kg^−1^ and a high specific power of, e.g., 8 kW kg^−1^ without specific energy loss at a high voltage up to 3.0 V. An ultralow performance fading of, e.g., ≈ 0.13% per 1000 cycles (90%–75,000 cycles) outperforms previously reported results. Also, the enhanced charge transfer kinetics and reduced interfacial resistance at high current rates deliver high specific energy without compromising the high specific power along with high durability, and thereby bridge the batteries and capacitors. Thus, kinetically enhanced NHCs can be a trendsetter for the development of advanced energy storage devices requiring high energy and high power. The future prospects are promising, as most technical and product indexes, including the cycling stability, rate capabilities, weight loading, and cost of rechargeable SIBs and NHCs, have been raised to a commercially acceptable level as competitive alternatives to LIBs or capacitors. The Ragone plot of NaTi_2_(PO_4_)_3_-based non-aqueous SIBs, aqueous SIBs, and sodium-ion hybrid capacitors is shown in Fig. 1b.

A systematic overview of the emerging critical progress is an urgent necessity. In this review, we will cover the recent progress in NTP-based electrodes for both NIBs and NHCs. The underlying synthesis methods, materials modification strategies, and electrochemical properties will be summarized in detail. Further, the major challenges and perspectives regarding the prospects for the use of NTP-based electrodes in energy storage systems will be summarized.

## Sodium-Ion Batteries

NASICON-structured NaTi_2_(PO_4_)_3_ has attracted increasing attention as a promising anode material due to the specific “zero-stress” framework with high Na-ion conductivity, long-term cycling stability, excellent rate capability, large theoretical capacity (133 mAh g^−1^) as well as low cost, environmentally benignity, and much better safety characteristics [25, 43–49]. The reversible (de)sodiation between NaTi_2_(PO_4_)_3_ and Na_3_Ti_2_(PO_4_)_3_, i.e., the redox reactions of Ti^3+^ ↔ Ti^4+^ via a two-phase reaction mechanism, can be represented by Eq. (1) [44, 45]:

1 $${\text{NaTi}}_{2} \left( {{\text{PO}}_{4} } \right)_{3} + 2{\text{Na}}^{ + } + 2{\text{e}}^{ - } \leftrightarrow {\text{Na}}_{3} {\text{Ti}}_{2} \left( {{\text{PO}}_{4} } \right)_{3}$$

During discharge, the Na atom on Na_2_ sites is reversibly removed while the Na_2_ sites remain unaffected.

### Non-aqueous Batteries

Delmas et al. first reported the reversible sodiation of NaTi_2_(PO_4_)_3_ in an organic electrolyte and revealed that two Na^+^ ions could be reversibly intercalated to form Na_3_Ti_2_(PO_4_)_3_ via a two-phase mechanism [50] showing a pair of typical well-defined redox peaks at 2.2/2.0 V within the potential window of 1.6–2.6 V (vs. Na/Na^+^) [44], and a number of research groups conducted a detailed structural elucidation of the electrochemical transition and structural control related to this compound [51–56]. To enhance the inherent low electronic conductivity of the phosphate framework especially for high-power SIBs application, numerous efforts have been made to improve its electrochemical performance by nanoarchitecturing the NTP particles and incorporating conductive carbon coating/networks (e.g., amorphous carbon, CNTs, or graphene) [45, 48, 57–63].

#### Nanoarchitectures of NTP

**Fig. 2:**
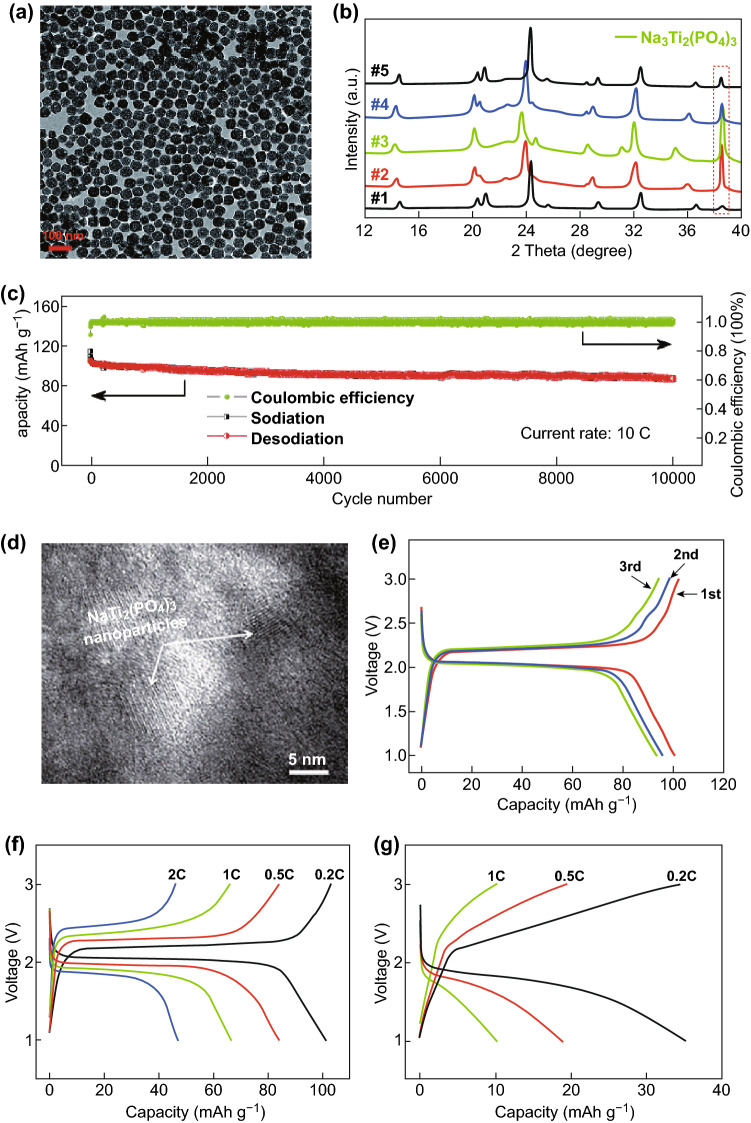
a TEM image of typical NTP nanocubes obtained via a one-pot solvothermal method. **b** XRD diffraction patterns recorded at different stages of NTP upon charging/discharging. The diffraction peak marked with a dark-wine-red-dashed box in **b** corresponds to the aluminum current collector. **c** Cycling performance of NTP electrodes obtained at current rates of 10 C [48]. Copyright 2015, the Royal Society of Chemistry. **d** HRTEM images of the mesoporous NTP/CMK-3 nanohybrid electrode after 1000 cycles at the rate of 0.5 C. **e** The first three charge–discharge profiles of the NTP/C electrode at 0.2 C. **f** Galvanostatic charge–discharge (GCD) plots of NTP/C and **g** pure NTP anodes at various C-rates [58]. Copyright 2014, the Royal Society of Chemistry. (Color figure online)

Nanostructured NTP with higher surface areas usually has a higher capacity; e.g., it was reported by Niu and his coworkers that NTP nanoparticles synthesized by a pyro-synthetic reaction in comparison to that by a traditional solid-state method show a much higher surface area and better electrochemical performance: the rapid pouring of the homogeneous starting precursors onto the hot plate resulted in rapid precursor decomposition and subsequent nucleation of nanoparticles [64]. Yang et al. synthesized a high-rate non-aqueous SIB anode material of porous NTP nanocubes with an over 10,000-cycle lifespan (75.5% retention of the initial capacity at a 10 C rate) (Fig. 2a, c). The controllable synthesis of porous NTP nanocubes with high regularity and porosity via a one-pot solvothermal route through the long-range oriented attachment of tiny nanocrystals into the 3D architectures (i.e., oriented attachment growth) will enrich the NTP system and provide several possible candidates for promising SIB anodes. The as-synthesized porous nanocubes showed excellent high rate performance and could still deliver considerable reversible capacities after deep charging/discharging over 15,000 cycles at an extremely high rate of 100 C. Ex situ TEM and XRD analyses further supported the conclusion that the electrochemical performance of NTPs is completely reversible upon Na^+^ intercalation (Fig. 2b), ensuring robust structural stability and long cycle life for practical applications [48].

Crystalline order or the degree of crystallinity in NTP also plays an important role in electrochemical properties such as capacity and electrode kinetics. Ko et al. studied the correlation of electrochemical performance with crystalline order. Starting with an amorphous NTP powder prepared by the Pechini method, varied NTP nanoparticulates of different degrees of crystallinity were derived via calcination. It was observed that poorly crystalline NTP samples (derived at 500–600 °C) exhibited low specific capacities and broad voltammetric features for Na^+^ insertion, characteristic of surface-limited processes; and highly crystalline NTP samples (derived at 700–800 °C) with the well-formed NASICON structure exhibited sharp voltammetric peaks and diffusion-limited kinetics in both organic (i.e., non-aqueous) and aqueous electrolytes. Further integration of nanocrystalline NTP with conductive networks can enhance the local electronic conductivity to a theoretical specific capacity in a non-aqueous electrolyte and an adequate capacity in a mildly aqueous electrolyte with significantly improved long-term stability [65].

#### Nanolayer-Coated NTP

Methods for the incorporation of carbon with NaTi_2_(PO_4_)_3_ vary from carbon layer coating [60, 66–70], electrospinning [71–76], solvothermal synthesis [49, 62, 77], pyro-synthesis [64, 78], spray-drying [74], and assembly [79] to ball milling [80] for different morphologies or structures, e.g., porous plates [77], 1D nanofibers [71, 72], nanocubes [49, 73], mesoporous materials [67, 73], and hierarchical nanocomposites [60, 79]. Some related works about carbon-coated architectures have reported excellent or enhanced performance; e.g., the nanosized porous carbon-coated NTP particles prepared by He et al. through a hydrothermal process combined with various carbon coating steps showed superior rate (capacities of 106 mAh g^−1^ at 10 C over 1000 cycles, 111 mAh g^−1^ at 30 C) and low-temperature properties (98 mAh g^−1^ at 10 C and 61 mAh g^−1^ at 20 C even at − 20 °C). This indicated that the addition of a small amount of Na_3_Ti_2_(PO_4_)_3_ (NVP) intermediate powder accounts for the in situ catalytic formation of more *sp*^2^-type carbon coating, i.e., highly graphitic carbon (graphene-like layers) coating, for excellent electrochemical performance of high-power SIBs [73].

Zhang et al. synthesized an open holey-structured framework for an NTP/C nanocomposite with open channels in nanocube morphologies for faster Na-ion transport by using a solvothermal reaction followed by pyrolysis. It demonstrated fast Na-ion transport and preferable battery performance, with a very small capacity decrease from 124 to 120 mAh g^−1^ in the wide range 0.5–50 C. An excellent discharge capacity of 103 mAh g^−1^ (88.3% retention of the first cycle) was delivered after an ultralong lifespan of 10,000 cycles at a super-high rate of 50 C [49]. Pang et al. synthesized a mesoporous NaTi_2_(PO_4_)_3_/CMK-3 (NTP/C) nanohybrid with high-crystallinity NTP nanoparticles (size of ~ 5 nm) homogeneously embedded in the highly conductive mesoporous CMK-3 matrix via a solvothermal route followed by calcination. The CMK-3 not only served as a rigid, interconnected conductive support but also suppressed agglomeration or overgrowth of NTP nanoparticles. Even over 1000 cycles, the nanohybrid showed an integral structure with a well-crystallized rhombohedral NaTi_2_(PO_4_)_3_ phase (Fig. 2d). Further, the nanohybrid anode showed some typical characteristics including a pair of well-defined sharp and stable redox peaks (located at 2.09 and 2.16 vs. Na^+^/Na corresponding to the redox reaction of Ti^4+^/Ti^3+^ during the reversible insertion–extraction reaction of Na^+^ in the NTP lattice), high initial charge–discharge capacities (corresponding to a 75% utilization of its theoretical capacity with a high coulombic efficiency (CE) of 98% in the potential voltage window 1–3 V), and enhanced rate capabilities (with distinct charge–discharge voltage plateaus) compared to pure NTP at the same rate (Fig. 2e–g), which can be attributed to the fast Na^+^ insertion–extraction kinetics and good electrical conductivity of the mesoporous hybrid architecture [58].

**Fig. 3:**
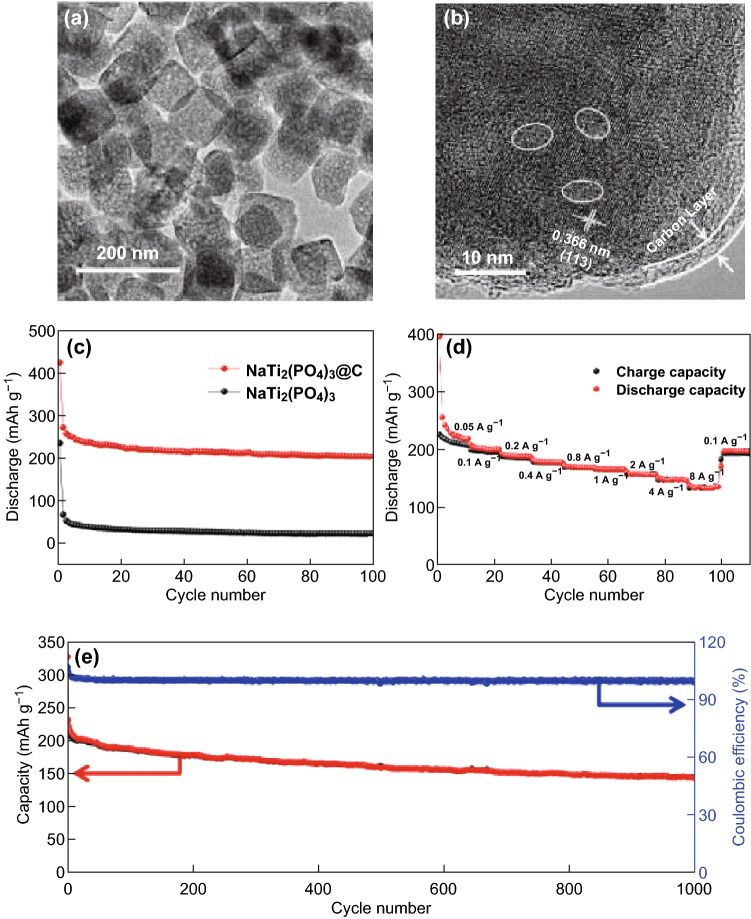
**a**, **b** TEM images of NaTi_2_(PO_4_)_3_@C. **c** Cycling performances of NaTi_2_(PO_4_)_3_@C and NaTi_2_(PO_4_)_3_ at 100 mA g^−1^. **d** Rate capabilities of NaTi_2_(PO_4_)_3_@C and **e** cycling performance of NaTi_2_(PO_4_)_3_@C at the higher current density of 1 A g^−1^ [81]. Copyright 2017, Elsevier Ltd

**Fig. 4:**
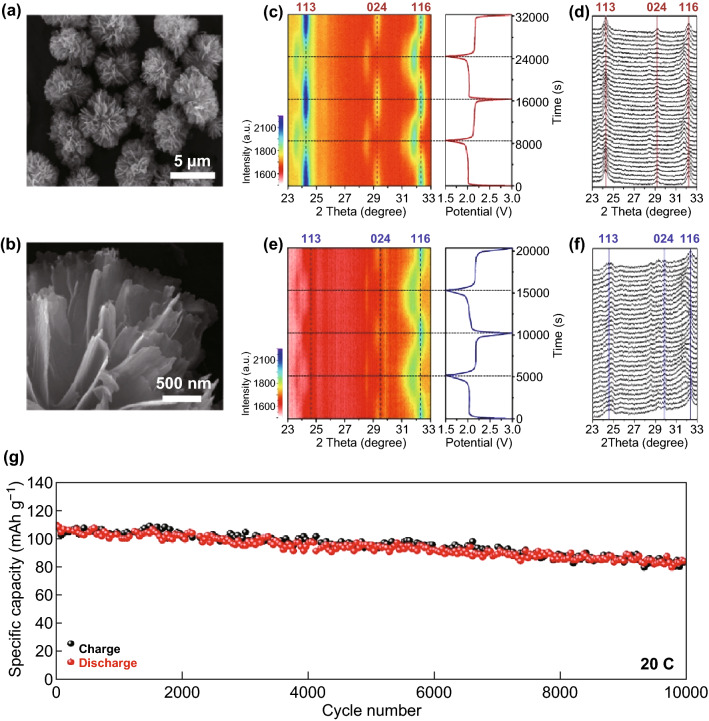
**a**, **b** Field emission SEM images of carbon-coated hierarchical NaTi_2_(PO_4_)_3_ mesoporous microflowers (NTP/C-F), and in situ XRD patterns of the carbon-coated NTP particles (NTP/C-F and NTP/C-P)) half-cells during galvanostatic discharge and charge at 50 mA g^−1^ and a voltage range of 1.5–3.0 V. **c** image plot of diffraction patterns and **d** selected individual diffraction patterns of the NTP/C-F stacked against the voltage profile at 23–33° during the first two discharge–charge cycles. **e** Image plot of diffraction patterns and **f** selected individual diffraction patterns of the NTP/C-P stacked against the voltage profile at 23–33° during the first two discharge–charge cycles. **g** Ultralong cycling stability of the NTP/C-F for 10,000 cycles at 20 C [41]. Copyright 2016, Elsevier Ltd

Ma and coworkers prepared porous NaTi_2_(PO_4_)_3_@C nanocubes (Fig. 3a, b) via a hydrothermal route followed by carbon coating using oleic acid as the carbon source. When utilized as an SIB anode, the mesoporous nanocubic hybrid could deliver an enhanced capacity of 201 mAh g^−1^ at 100 mA g^−1^ after 100 cycles, high rate capabilities, and a long cycle capacity of 140 mAh g^−1^ at 1.0 A g^−1^ over 1000 cycles in the voltage window 0.01–3.0 V (Fig. 3c–e) [81]. The extended work voltage below 1.0 V of the multi-step process, corresponding to Ti^3+^ ↔ Ti^2+^, can be described by Eq. (2) [51, 69, 81]:

2 $${\text{Na}}_{ 3} {\text{Ti}}_{2} \left( {{\text{PO}}_{4} } \right)_{3} + {\text{Na}}^{ + } + {\text{e}}^{ - } \leftrightarrow {\text{Na}}_{ 4} {\text{Ti}}_{2} \left( {{\text{PO}}_{4} } \right)_{3}$$

which theoretically contributes an additional specific capacity of 119 mAh g^−1^ (i.e., with a total theoretical capacity of up to 252 mAh g^−1^ for Na_3_Ti_2_(PO_4_)_3_ in the voltage window 0–3 V vs. Na^+^/Na) with a pair of primary redox peaks at a voltage as low as approximately 0.44 V [78], although in the first discharge process, a solid electrolyte interphase (SEI) will form below 1.5 V via consumption of extra Na^+^ in the electrolyte, and the cyclability may deteriorate [69, 81–83]. Mai and coworkers designed an architecture comprising carbon-coated hierarchical NTP mesoporous microflowers (Fig. 4a, b) via a facile and controllable solvothermal route followed by annealing. The unique structure endows the hierarchical composite with outstanding structural stability, enhanced charge transfer kinetics, and suppressed polarization. It demonstrated a superior Na storage performance with a high initial capacity of 125 mAh g^−1^ at 1 C, an excellent rate capability of 95 mAh g^−1^ that is high even up to 100 C, and an ultralong cycling stability (77.3% capacity retention, i.e., 85 mAh g^−1^, even after 10,000 cycles at 20 C) (Fig. 4g). A time-resolved in situ X-ray diffraction study (Fig. 4c–f) reveals the typical two-phase electrochemical reaction with reversible structure change during sodiation/desodiation. The superior performance can be ascribed to the synergistic effect of the carbon skeleton for structural stability, as well as to the highly stable and open framework with rich mesopores for intimate contact between the electrolyte and active nanosized NTP of rapid Na-ion diffusion and electron transport [41].

**Fig. 5:**
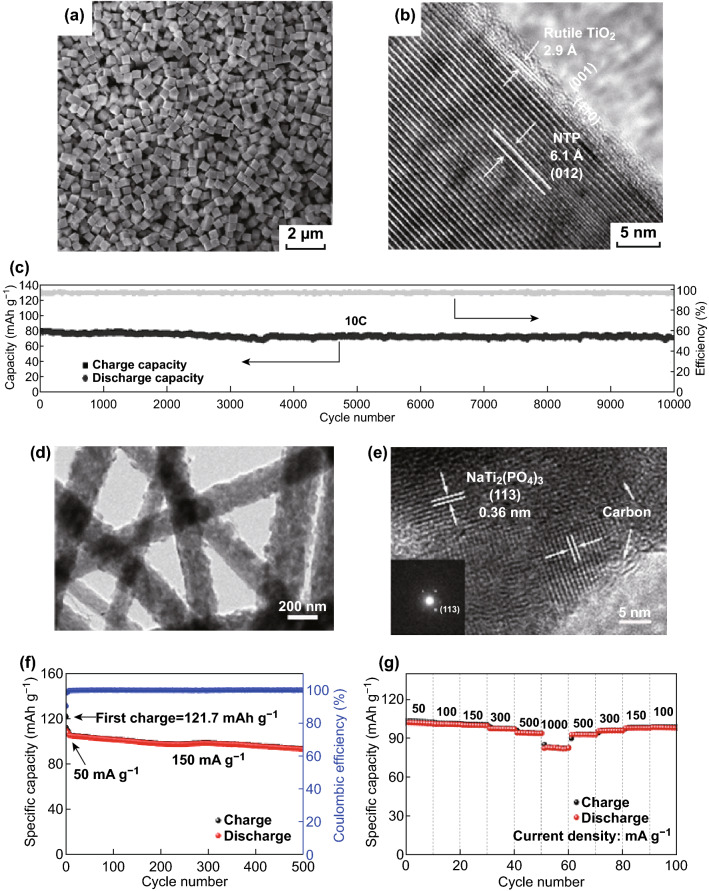
**a** SEM image and **b** HRTEM image of NTP-RT at the edge and **c** ultralong-term cycling performance at a high current density of 10 C [84]. Copyright 2015, Wiley–VCH Verlag. **d, e** TEM images at different magnifications. **f** Cycling stability and **g** various charge/discharge rates of NTP/CNFs//NiHCF full cell [75]. Copyright 2018, American Chemical Society

Apart from carbon, TiO_2_ has also proved to be an efficient coating layer for NTP. Yang et al. [84] designed a type of highly regular and single-crystalline NTP nanocubes (average diameter ca. 500 nm) with a synergistic nanocoating of carbon and rutile TiO_2_ (C/NTP-RT) (Fig. 5a, b). The amorphous carbon layer (thickness ~ 10 nm and carbon content of ~ 4.2 wt%) was coated on NTP-RT to enhance the electronic conductivity for higher rate capability. When applied to an SIB anode, the C/NTP-RT exhibited capacitor-like superb rate performance and battery-like high capacity and ultralong-life feature; e.g., over 10,000 cycles, it could deliver a high rate capacity of 72.3 mAh g^−1^ with a capacity retention of 89.3% (of the initial 83.5 mAh g^−1^) at 10 C (Fig. 5c). This facile one-step hydrothermal method of TiO_2_ precursor addition provides a new strategy for optimizing electrode materials and may be utilized in future high-rate, ultra-stable, and low-cost energy storage applications.

Embedding the NTP nanoparticles in the nanocarbon networks will considerably enhance the Na-ion/electron transfer for highly reversible and ultrafast sodium storage [79, 85–88]. For example, by using a simple soft-template method, Yu and coworkers designed an NTP/C composite with nanosized NTP particles coated by a thinner carbon shell and interconnected by a carbon network. With the synergistic effects of a lower charge transfer resistance and a larger surface area for the electrolyte to soak in and sufficient void to buffer the volume variation during the repeated Na^+^ insertion/extraction, the anode materials demonstrated outstanding rate performance (108 mAh g^−1^ at 100 C; i.e., a discharge/charge time of 36 s) and long cycle life (83 mAh g^−1^ at 50 C over 6000 cycles), as well as a lower polarization and higher initial CE (ICE; e.g., ~ 98% at 1 C) [85]. Similar to amorphous carbon networks, carbon nanotubes are also a superior framework for enhancing Na^+^/e^−^ conductivity. Xu et al. fabricated a hierarchical porous nanocomposite architecture consisting of MWCNT-threaded mesoporous NTP nanocrystals for high-performance sodium electrodes. With a high ICE of 99%, high rate capability of 74 mAh g^−1^ at 50 C, and long-term cycling stability (74 mAh g^−1^ after 2000 cycles at 10 C) superior to that of the physically mixed reference composite, it provides a general hetero-assembly approach to various types of nanocomposites for high-performance SIBs [79]. Wang et al. [86] designed a carbon-nanotube-decorated NTP/C nanocomposite with high rate performance; especially impressive is that the composite could exhibit low-temperature (− 20 °C^)^ performance with a capacity of 65 mAh g^−1^ at 10 C. Wei et al. fabricated porous NaTi_2_(PO_4_)_3_/C hierarchical nanofibers (Fig. 5d, e) via an electrospinning method followed by annealing. The NTP/CNFs as an anode for SIBs exhibited a high reversible capacity of 120 mAh g^−1^ at 0.2 C, high rate capability (71 mAh g^−1^ at 20 C), and long cycling stability similar to those of sodium-ion full cells and hybrid sodium-ion capacitors. When assembled using nickel hexacyanoferrate (NiHCF, Na_4_Fe(CN)_6_) as cathode material, it showed a high ICE of ~ 90% (corresponding to initial charge and discharge capacities of approximately 122 and 110 mAh g^−1^) and a capacity retention of ~ 90% after 500 cycles at 150 mA g^−1^ with a CE that approached 100% as well as excellent rate capabilities when operated between 0.5 and 2.5 V (Fig. 5f, g) [75]. Yu et al. prepared a similar structure of ultrafine nanoparticles encapsulated in 1D N-doped carbon nanofibers and extended the voltage window to 0–3.0 V. The poor electrical conductivity of NTP was significantly improved, and the composite demonstrated stable and ultrafast Na storage capability with a specific capacity of 121 mAh g^−1^ at 10 C after 2000 cycles and 105 mAh g^−1^ after 20,000 cycles, as well as superior rate performance from 0.2 to 20 C with a recovery efficiency of 99.4% [76].

Full cell batteries with high energy and long life cycle remain a significant challenge, and the formation of an SEI on both cathodes and anodes (especially for the hard-carbon-incorporated composites) has revealed a potential way to realize long-term stability of SIB full cells. A pre-cycling of cathodes and anodes leads to pre-formation of an SEI, which mitigates the additional consumption of Na^+^ ions in full cells for higher ICE as well [89–94]. In addition, the adoption of Al (compared to the more common choice, Cu) as the anode current collector in NASICON NTP//NVP full cells will enhance the specific energy, although some improvements are needed to achieve better power capability and energy efficiency [95]. The simultaneous optimization of the structural stability of cathode materials will further enhance the cycle performance of the Na-ion full cells [96].

#### 2D NTP Composites

**Fig. 6:**
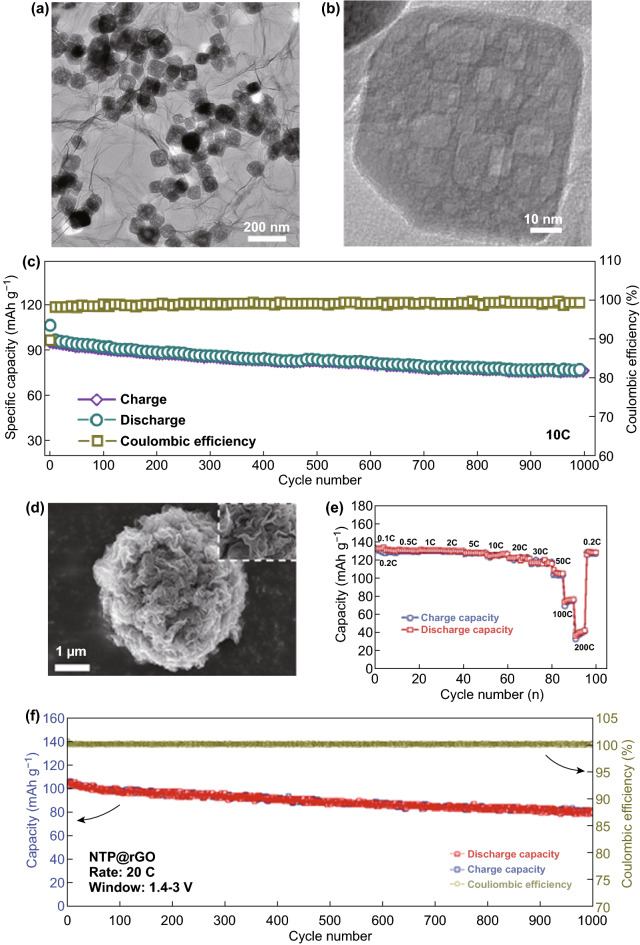
**a** TEM and **b** HRTEM images of NTP ⊂ GN and **c** long cycling performance of the NTP ⊂ GN electrodes. Note that all the capacity values for the NTP ⊂ GN electrodes are calculated on the basis of the mass of NTP, which is 65% of the whole electrodes including NTP, graphene, carbon black, and PVDF [45]. Copyright 2015, American Chemical Society. **d** SEM images showing a 3D graphene network obtained by removing NaTi_2_(PO_4_)_3_ nanoparticles with HF solution. **e** Rate capability of the NTP@rGO electrode. **f** Long-term cycling performance of the NTP@rGO electrode at a high current rate of 20 C over 1000 cycles in the voltage window 1.4–3.0 V [44]. Copyright 2016, Wiley–VCH

Two-dimensional (2D) graphene and its analogs are an ideal conductive matrix for electrochemical applications owing to its excellent electrical conductivity, high specific surface area, and mechanical robustness [27, 63, 97–101]. The Yu group synthesized a novel architecture of porous NTP nanoparticles embedded in 3D graphene networks (NTP ⊂ GN) (Fig. 6a, b) via a self-assembly and post-heat-treatment route. By synergistically combining the advantages of a 3D graphene network and 0D porous nanoparticles, the architecture significantly facilitates the electron/ion transport kinetics and ensures electrode structure integrity, leading to excellent electrochemical performance as reflected by the high rate capability (112, 105, 96, 67 mAh g^−1^ at 1, 5, 10, 50 C, respectively), long cycle life (80% capacity retention over 1000 cycles at 10 C), and a high ICE (> 79%) (Fig. 6c). The ultrafast and stable performance as a promising advanced SIB anode exceeds that of other hard carbon or metal alloy materials, and is comparable to that of supercapacitors [45].

Fang et al. designed 3D-graphene-decorated NaTi_2_(PO_4_)_3_ microspheres (NTP@rGO) (Fig. 6d) via a hierarchical graphene-embedded process using a facile spray-drying method with post-calcination for superior high rate and ultracycle-stable sodium storage performance as a promising SIB anode [44]. The as-obtained NTP@rGO composite demonstrated a high reversible capacity of 130 mAh g^−1^ (close to 96% theoretical capacity) at 0.1 C (with almost equal values from 0.1 to 2 C; 1 C = 133 mA g^−1^), long stable cyclability (77% capacity retention over 1000 cycles at 20 C), and ultrahigh rate capability (38 mAh g^−1^ at 200 C) (Fig. 6e, f). When paired with Na_3_V_2_(PO_4_)_3_ as a cathode, the NTP@rGO//NVP/C full cell can deliver a high discharge capacity of 128 mAh g^−1^ at 0.1 C based on the anode mass, and an outstanding long-life cycling performance with 80% capacity retention over 1000 cycles and a CE of above 99.5%, as well as a high rate performance of 88 mAh g^−1^ at 50 C. The SIB full cell exhibited excellent specific energy and power densities that are superior to those of hybrid batteries and supercapacitors [102, 103], showing an energy density of 73 Wh kg^−1^ at a power density of 7.6 W kg^−1^ (0.1 C) and even maintaining 38.6 Wh kg^−1^ at a power density up to 3167 W kg^−1^ (50 C). The excellent properties can be attributed to the combined advantages of the graphene-coated nanosized NTP particles and the presence of the highly conductive 3D graphene network, which remarkably enhanced the ionic/electronic transport and buffered the volume variation during sodiation and desodiation. The novel method for 3D hierarchical spherical structures shows a promising alternative route for realizing superior SIBs.

**Fig. 7:**
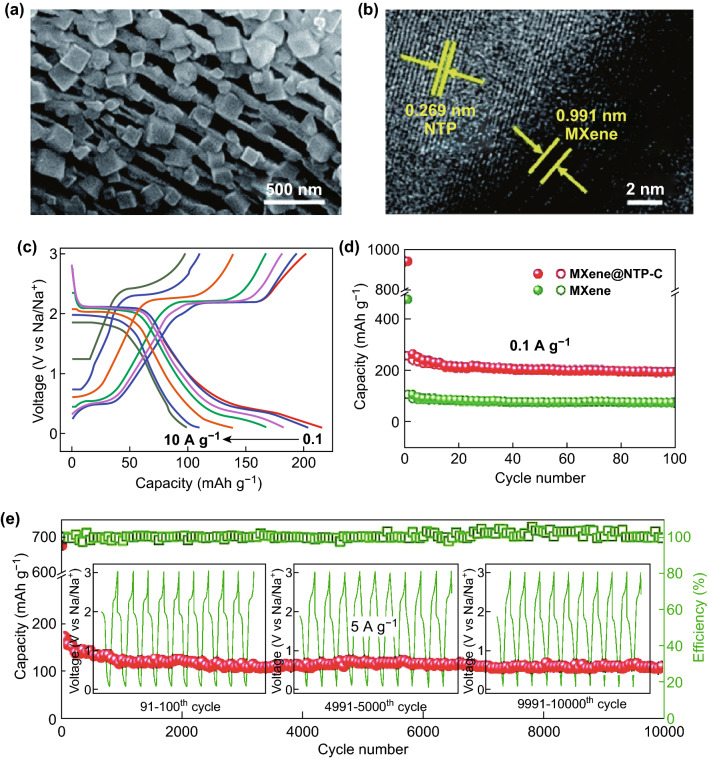
**a** TEM and **b** high-magnification TEM images of MXene@NTP-C. **c** Galvanostatic discharge/charge profiles of MXene@NTP-C at varied current densities of 0.1–10 A g^−1^. **d** Cycling performance of MXene@NTP-C and MXene at 0.1 A g^−1^. **e** Cycling performance of MXene@NTP-C at high current densities of 5 A g^−1^. The inset of **e** shows the voltage–cycle profile at three different stages [110]. Copyright 2018, the Royal Society of Chemistry

Some similar structures also showed excellent performance because the nanosized structures and the intimate contact between NTP and high-conductive graphene significantly reduced the transport lengths of the Na^+^ ions and the “expressway” of electron transport [61, 104, 105]. For example, a phase-pure NaTi_2_(PO_4_)_3_/reduced graphene oxide (rGO) nanocomposite, prepared using a microwave-assisted one-pot solvothermal method and post-heat treatment with well-crystallized and uniformly anchored NTP nanoparticles (30–40 nm) on an rGO matrix through Ti–O–C bonds, can exhibit a high specific capacity of 129 mAh g^−1^ (approaching the theoretical value) at 0.1 C and an excellent rate capacity (72.9% capacity retention at 50 C), as well as superior cycling performance with merely 4.5% capacity loss over 1000 cycles at a high rate of 10 C (the CE remained at 99.8%) without using any other additional conducting agent in the anode. Furthermore, the NTP/rGO nanocomposite showed excellent high-temperature cyclability up to 55 °C [106]. Ma and coworkers designed and developed a structure consisting of porous NaTi_2_(PO_4_)_3_ nanocubes (50–100 nm) anchored on porous carbon nanosheets (average thickness of ~ 10 nm) for high rate capability. With the extended potential window of 0.01–3.0 V, the open 3D framework structure exhibited a high initial discharge capacity of 485 mAh g^−1^ at 0.1 A g^−1^, and a high capacity of 98 mAh g^−1^ was retained at 4.0 A g^−1^ after 2000 cycles [107]. However, the low ICE needs to be improved for further practical application in full SIBs. In addition, by incorporation of 2D MXene nanosheets with inherent advantages including a suitable interlayer spacing for accommodating sodium ions, low working potentials, environmental benignity, and exceptional chemical durability [108–111], Zhi and coworkers designed a dual-mode sodium storage device based on the combination of pseudocapacitance-type and battery-type electrochemical behavior. The dual-mode anode material (MXene@NTP-C) (Fig. 7a, b) for SIBs showed superior rate capacities (208–102 mAh g^−1^ at 0.1–10 A g^−1^ within the voltage window 0.1–3.0 V vs. Na/Na^+^) as well as remarkable cycling performance up to 10,000 cycles (109 mAh g^−1^ at 5 A g^−1^) (Fig. 7c–e), which presents an opportunity to balance the energy and power densities [110].

#### Flexible or Binder-Free Electrodes

**Fig. 8:**
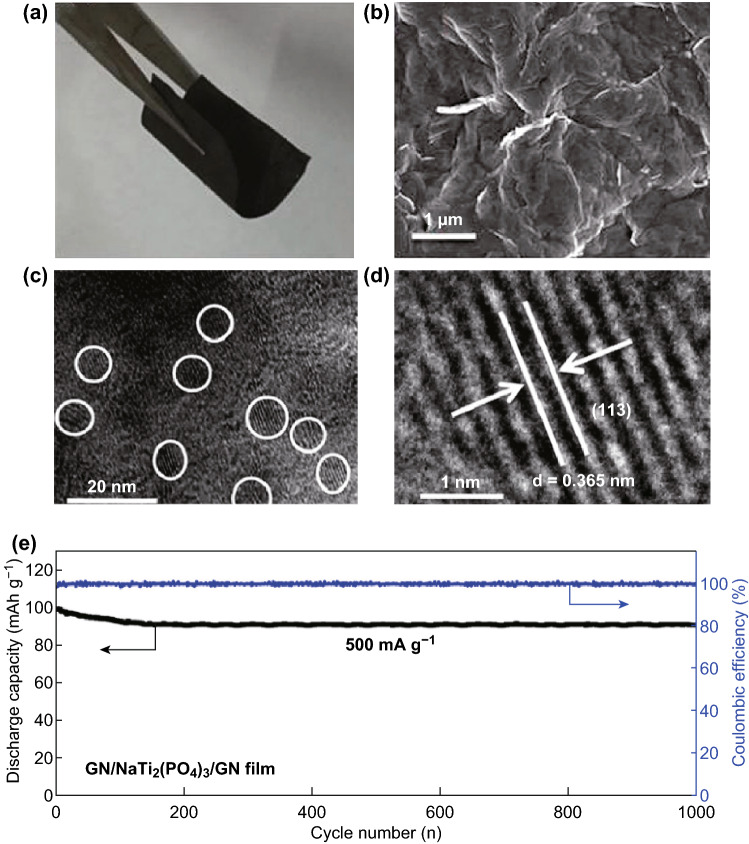
**a** Digital photograph and **b** FESEM images of the as-fabricated flexible GN/NaTi_2_(PO_4_)_3_/GN film. **c, d** HRTEM images of GN/NaTi_2_(PO_4_)_3_/GN film. **e** Long-term cycling stability of GN/NaTi_2_(PO_4_)_3_/GN film electrode at 500 mA g^−1^ [112]. Copyright 2018, American Chemical Society

Flexible energy storage devices are attracting considerable interest in next-generation bendable, wearable, or implantable electronic systems [112–118]. The Cao group constructed a flexible and binder-free sandwich-structured NaTi_2_(PO_4_)_3_ film electrode (Fig. 8a–d) with a two-step graphene hybridizing method for an ultra-stable and long-life SIB anode. The flexible anode with an interconnected framework exhibited excellent cycling stability for sodium half-cells (91% capacity retention over 1000 cycles at 500 mAh g^−1^) (Fig. 8e). When assembled into flexible full SIBs with Na_0.44_MnO_2_ as a cathode, it showed good cycling performance both under flat and bent states as well as a high ICE (82.3%) in the voltage window 2.0–4.0 V [112]. Previous work has reported the design of a synergistic flexible electrode, mesoporous NTP nanocrystals embedded in monolithic hierarchical porous carbon assembly rGO and CNTs for high-performance SIBs (maintaining a high reversible capacity of 125 mAh g^−1^ at 1 C, long cycling life of 5000 cycles at 10 C with 82% capacity retention, and high rate capability of 73 mAh g^−1^ at 30 C; superior to the rGO-only recipe) [114], as well as other self-supporting anodes such as mesoporous NTP nanocrystals confined in MWCNTs network (film thickness of 50 μm; showing a high volumetric/areal capacity: 132 mAh g^−1^ at 1 C with ICE of 99%, 62 mAh g^−1^ at 50 C, and long-term cycling stability with a capacity of 87% at 10 C after 3000 cycles) [119]. Yang and coworkers constructed another 2D nanocomposite architecture comprising mesoporous NTP nanocrystals, rGO, and thermally treated protein (TP) (denoted by MNTP-TP@rGO) as a free-standing electrode for an advanced SIB anode. The 3D interconnected carbon network of rGO and TP acts as a support for anchoring the well-distributed mesoporous NTP nanocrystals and also a current collector. As a free-standing anode for a half-cell, it delivered a high rate capacity of 52.8 mAh g^−1^ at 50 C and robust cycling stability (80% capacity retention over 1000 cycles at 5 C). When paired with Na_3_V_2_(PO_4_)_3_/C (NVP/C) as a cathode, the free-standing anode demonstrated a high specific capacity of 58 mAh g^−1^ with outstanding cycling stability (98% capacity retention over 100 cycles at 1 C) [113]. However, it should be noted that despite a flat voltage plateau, the voltage is only approximately 1.2 V lower than that of the full cell with Na_0.44_MnO_2_ as a cathode [112]. The strategy developed here can provide a general route for preparing other graphene-based flexible SIBs.

#### High-Safety SIBs

Development of high-safety and long-lifespan SIBs is urgently needed for large-scale energy storage applications. All-solid-state SIBs have attracted considerable attention for their safety and long-term durability [120–125]. Solid-state rechargeable SIBs based on ceramic (e.g., Na-βʺ-Al_2_O_3_) electrolyte with high sodium ion conductivity can demonstrate an extremely stable voltage plateau of ~ 2.1 V in the half-cell and an initial discharge capacity of 133 mAh g^−1^, although the cycling and rate performances may be improved via modification of interfacial incompatibility (or cell resistance and intrinsic polarization) compared to that of typical non-aqueous SIBs [126]. To further suppress the formation of Na dendrite, Goodenough and coworkers designed a NASICON ceramic electrolyte assisted by an in situ-formed thin interfacial interlayer or by the introduction of a dry polymer layer for high-temperature performance and safety advantages. The as-prepared all-state-batteries with NTP as the anode showed high cycling stability and CE (99.8 ± 0.02%) at 65 °C due to the enhanced wetting of sodium on the interfacial interlayer that suppresses dendrite formation and growth.

Currently, most batteries for electrical energy storage (EES) use highly flammable and volatile organic carbonate esters as electrolytes, which may cause severe safety problems, and an intrinsic system is in high demand, especially for large-scale EES applications [127]. Apart from aqueous electrolytes [128] and solid-state electrolytes, etc., [121, 126, 129], proton-type organic phosphonates have demonstrated promise for safer SIBs with a wide electrochemical window and high ionic conductivity [127, 130]. Cao and coworkers designed and constructed an interesting all-phosphate-based battery by using a NTP anode, Na_3_V_2_(PO_4_)_3_ cathode, and trimethyl phosphate (TMP) electrolyte for zero-strain SIBs with intrinsic safety, high rate performance, and long cycle life [127]. The full cells demonstrated good cycling performance (73.7% capacity retention over 1000 cycles) and promising designing flexibility for practical application. Soon after that, a similar self-standing all-phosphate SIB with high mass loading for fast cycling was realized [131].

For large-scale and low-cost energy storage, non-aqueous semi-solid flow batteries (SSFBs)—a special class of redox flow batteries (RFBs)—based on the rich chemistry of Na-ion intercalating compounds, e.g., the NTP anode and P2-type cathodes, may serve as an inspiration [132].

#### Trace-Element-Doped NTP Materials

As an effective method of improving the electrochemical performance of electrode materials, lattice doping has been proposed and widely used in LIBs and SIBs [133]. As for research on NTP-based materials, it was first demonstrated by Mouahid and coworkers [134] that the doping of Al is beneficial for improving the ionic conductivity of NTP and improves electrochemical performance. Tirado’s group proposed that the low content of iron doping did not change the lattice structure but could enhance the capacity values and improve capacity retention [135–137]. Goodenough’s group utilized the NASICON-structured Na_3_MnTi(PO_4_)_3_ as both the anode and cathode to construct an aqueous symmetric SIB with an operating voltage of 1.4 V, stable cycle performance, and excellent rate capability [138]. Dai’s group demonstrated that Sn doping on the Ti site shows no obvious effect on the lattice structure and morphology of NaTi_2_(PO_4_)_3_/C but is very beneficial for improving the electrochemical properties of the NaTi_2_(PO_4_)_3_/C anode for aqueous LIBs [139]. Zhang and coworkers reported the synthesis of porous Na_3_MgTi(PO_4_)_3_ aggregates with a sol–gel method. The good rechargeable capacity of 54 mAh g^−1^ and better capacity retention performance (94.2% after 100 cycles) of Na_3_MgTi(PO_4_)_3_ compared to those of NTP demonstrate that the incorporation of electrochemically inert Mg^2+^ ions could improve the structural stability of Na storage materials and enhance cycling performance [140].

### Aqueous Batteries

Aqueous rechargeable alkali-ion (e.g., Li, Na, Mg-ion) batteries that do not employ costly, highly toxic, and flammable organic solvents were first reported by Dahn’s group in 1994 using VO_2_/LiMn_2_O_4_ electrodes and LiNO_3_ aqueous solution as the electrolyte. Because of the much higher ionic conductivity of the aqueous electrolyte compared to that of organic electrolytes, aqueous batteries always feature high round-trip efficiency and energy density and have attracted interest as possible substitutes for conventional non-aqueous rechargeable systems. However, the narrower stable voltage window and the poor cycling life of aqueous electrolytes compared with those of organic electrolytes prompted researchers to further develop high-performance aqueous rechargeable alkali-ion batteries [43, 141, 142]. Of equal or greater interest, due to the high natural abundance of sodium, low lost, and safety advantages for large-scale or stationary energy storage, are aqueous sodium-ion batteries (ASIBs) or aqueous rechargeable sodium batteries (ARSB) [19, 43, 128, 143–145]. Okada and coworkers first presented a working demonstration of the aqueous Na-ion full cell using an NTP anode [43, 47] and revealed that an aqueous electrolyte showed a higher conductivity and lower viscosity compared with those of non-aqueous (i.e., organic) electrolytes, as well as much smaller interfacial activation energy or higher kinetics of Na-ion transfer than those of Li-ion secondary batteries, which are advantages for high rate capabilities [47, 57, 146, 147].

**Fig. 9:**
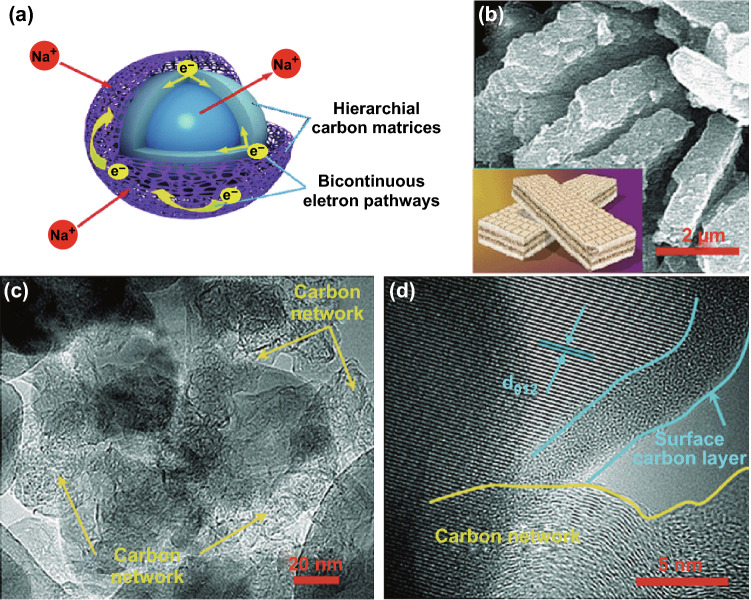
**a** Schematic image of enlarged parts in the wafer-like architecture with bicontinuous pathways for high-efficiency electron/ion transport. **b** SEM images of the wafer-like particles. **c** TEM image of the wafer-like particle. **d** HRTEM image of one primary particle decorated by hierarchical carbon [155]. Copyright 2015, the Royal Society of Chemistry

However, NTP suffers from considerable capacity fade when cycled slowly and deeply‚ and this could be attributed to the increasing pH caused by the presence of OH^−^ ions when water was reduced by Ti(III) ions at the surface of the electrode material and the following alkaline oxidation of the carbon conductive additive to carbonate ions at potentials lower than − 1.38 V versus Hg/Hg_2_SO_4_, which finally causes loss of electrical contact and ultimately failure of the cell [148]. Some studies reveal that the formation of amorphous transition metal phosphate layers and/or insoluble titanium sulfate phases on the surface of NTP particles, which are ionically and electronically insulating, via dissolution of sodium and titanium cations and hydrolysis of surface groups, may block the electrolyte access pathways to the electroactive particles embedded in the electrode and lead to capacity fade, although a deeper understanding of ‘‘phosphate–aqueous solution interface’’ reactions requires further investigations [149]. Upon optimizing the design and synthesis of nanostructured and higher conducting NTP composite anodes (e.g., NTP/C [60, 62, 150–152], NTP@C/Ag [153], NTP/conducting-polymers [148], or doped NTP [154]), the Na-ion diffusivity and electronic conductivity limitations encountered can be removed as well in organic electrolyte systems [43]. These composite structures include a self-assembled hierarchical carbon-decorated wafer-like 3D porous NTP/C composite with bicontinuous electron transport pathways via a surface nanoscale carbon layer and a porous microscale carbon matrix over NTP particles (Fig. 9a–d) (rate capability of 63 mAh g^−1^ high up to 50 C, and a stable capacity of 92 mAh g^−1^ over 300 cycles at 2 C with CE nearly 100%) [155]; a frogspawn-inspired hierarchical NTP/C (core–shell) array (rate capability of 78 mAh g^−1^ at 60 C, capacity retention of 88% at 1 C over 400 cycles and 89% at 20 C over 2000 cycles with a CE of nearly 100%) [156]; an NTP/C composite synthesized by a modified Pechini method and pyrolysis treatment, delivering an initial capacity of 129 mAh g^−1^ and maintaining 117 mAh g^−1^ over 50 cycles at 2 C and a high rate capability of 66 mAh g^−1^ at 20 C [150]; an NTP/CNTs/graphite composite in situ-synthesized with intermixed “intimate carbons”, showing improved performance (82 mAh g^−1^ over 100 cycles at 1 C with CE > 99.7%) over that with post-synthesized or with individual carbon [157]; and other composites with commercial carbon black or graphite [151].

Generally, the incorporation of 3D-carbon-based porous frameworks with high conductivity, high surface area, high structural stability, and good electrolyte penetration has attracted particular attention and exhibited high-efficiency electro/ion transport and better performance, and is thus considered an effective strategy for fabricating high-performance ASIBs [155]. Graphene, as a new and ideal 2D carbon material, has attracted intensive attention for application in energy conversion and storage due to its superior electrical conductivity, high surface area, structural flexibility, etc. The incorporation of graphene to form NTP composites or hybrids as a highly electronically conductive network improved cycling stability and rate performance, as was recently reported [59, 97]. For example, Zhang and coworkers prepared a 2D hybrid nanoarchitecture of NTP/graphene with highly crystalline NTP nanoparticles homogeneously anchored on the surface of conducting graphene nanosheets via a solvothermal method followed by calcination. The nanocomposite (with only 3.4 wt% of graphene) used as anode materials for ASIBs exhibited excellent electrochemical performance with high rate capabilities (110, 85, 65, and 40 mAh g^−1^ at 2, 5, 10, and 20 C, respectively) and a good cycling stability with 90% retention of the initial capacity over 100 cycles at 2 C. The remarkable improvement in specific capacity, rate performance, and cycling stability can be ascribed to the unique structures and the merits of both ingredients [59].

Apart from carbon components, a TiN layer was also applied on the surface of NTP to enhance the electronic conductivity in an aqueous electrolyte system. Zhang and coworkers synthesized a TiN-coated NaTi_2_(PO_4_)_3_ as an anode material for aqueous SIBs via a solvothermal routine and a subsequent nitriding process (i.e., calcination in ammonia gas). The optimized TiN-tailored NTP particles showed an improved rate capability and cycling performance with an initial capacity of 132 mAh g^−1^ and maintained 92 mAh g^−1^ after 100 cycles at 2 C, a large improvement over the pristine phase [158].

#### Full Cells

When combining with the cathodes, e.g., layered NaMnO_2_ [159], Na-birnessite with crystal water [160], alkali-cation-incorporated δ-MnO_2_ [161–163], Prussian-blue-type Na_2_CuFe(CN)_6_ [164], Na_2_NiFe(CN)_6_ [46], tunnel-structured Na_0.44_MnO_2_ (Na_4_Mn_9_O_18_) [43, 80, 152, 165, 166], NASICON-structured Na_3_V_2_(PO_4_)_3_ [167], and Na_2_VTi(PO_4_)_3_ [168], the aqueous SIBs exhibited high energy density and good cycle stability, which are particularly attractive for stationary energy storage applications. The use of Na-deficient NTP as an anode and Na-rich cathodes in an aqueous electrolyte system may be visually depicted as a “rocking-chair-type” SIBs [46]. In the full cells of an NTP system, it is generally agreed that the low electronic conductivity of the NTP anode was rate limiting, and by eliminating this limitation via NTP/C composite optimization, for example, ultrafast rate capability and superior high rate cycling stability can be obtained [80, 157, 165]. Chiang and coworkers demonstrated an ultrafast rate (> 100 C) and superior high rate cycling (> 1500 cycles) for aqueous NaTi_2_(PO_4_)_3_/Na_0.44_MnO_2_ (NTP/NMO) cells with a specific volumetric energy density of up to 127 Wh L^−1^ from the materials-only level, and a cell level density of ~ 65 Wh L^−1^ may be expected, which exceeds the energy density of more fully developed active carbon (AC)/NMO systems. The NTP–C nanocomposite synthesized by ball milling a 2–3% pyrolytic carbon from the glucose precursor accounts for the superior performance [43]. Zhang and coworkers further studied an NTP/MWCNTs–Na_0.44_MnO_2_ system to improve the electronic conductivity [165]. Thus, aqueous NTP/NMO may become a candidate for safe, low-cost, and high-power storage systems. However, possible causes for low-rate capacity fade may recur due to some complicated side reactions, e.g., partial diffusion of electrode materials, oxidation of the anode in its sodium-inserted state by dissolved oxygen or oxygen generated via water hydrolysis, or oxidation of the aqueous electrolyte by the charged cathode [43, 47]. For flexible aqueous SIBs, Guo et al. designed and fabricated a family of safe flexible SIBs as potential wearable or even implantable electronic devices based on a nanosized NaTi_2_(PO_4_)_3_@C anode, a Na_0.44_MnO_2_ cathode, and various Na^+^-containing aqueous electrolytes (including Na_2_SO_4_ solution, normal saline, or cell-culture medium), compared to that of either toxic flammable organic solutions or strong acid/base as electrolytes. The as-prepared belt- and fiber-shaped ASIBs exhibited excellent electrochemical performance (with high volumetric energy and power density, and long life) as well as high flexibility. This fiber-shaped electrode system also exhibited electrochemical deoxygenation and pH-changing features, which might be further applied in biological and medical fields [117].

Cathodes with different potential plateaus often influence the output voltage of the ASIB system [169]. For example, the Prussian-blue-type Na_2_NiFe(CN)_6_ combined with an NTP anode can deliver an average output voltage of 1.27 V, as well as an energy density of 42.5 Wh kg^−1^ and a capacity retention of 88% over 250 cycles at a 5 C rate [46]. Furthermore, adjustment of the transition metal cations at the M site in the Prussian blue compounds Na_*x*_M_*y*_Fe(CN)_6_ enabled the Na_2_CuFe(CN)_6_ cathode in the same ASIB system to exhibit a higher output voltage of 1.4 V (with a well-defined discharge plateau and a slight decrease from 1.4 to 1.1 V) as well as an enhanced energy density (48 Wh kg^−1^), rate, and cycling performance [164]. The NASICON-type Na_3_V_2_(PO_4_)_3_ assembled aqueous full cell with NTP as the anode demonstrated a flat discharge plateau at 1.2 V and could maintain a high rate performance (58 mAh g^−1^ at 10 A g^−1^) well, showing a high energy density of 29 Wh kg^−1^ at a power density of 5145 W kg^−1^ [167]. Layered NaMnO_2_ and its full cells with an NTP anode delivered an inclined curve of the voltage profile (e.g., 1.8–0.5 V) without plateaus, as well as an energy density of 30 Wh kg^−1^ at a power density of 50 W kg^−1^ and could retain 75% of the initial capacity over 500 cycles at 5 C [159]. The tunnel-structured Na_0.44_MnO_2_ full cell coupled with NTP showed a distinct flat plateau and an average operation voltage of 1.13 V, slightly lower than that of a typical commercial battery such as the Ni–Cd battery (1.2 V), but an initial reversible specific capacity of 85 mAh g^−1^ [80] or a higher capacity of 114 mAh g^−1^ [155] could be obtained. Through further modification, the Ti-substituted Na_0.44_MnO_2_ full cell coupled with an NTP anode could exhibit an average operating voltage of 1.2 V, higher rate capabilities (54 mAh g^−1^ at 10 C), and much more stable cycling performance (76 mAh g^−1^ at 2 C with a very small capacity decay up to 300 cycles) for practical applications [166].

### Electrolyte Dependence of Performance

Various electrolytes have currently been used and developed for non-aqueous SIBs based on the rationale for specific choices regarding cell setup and usage conditions [129]. In regard to aqueous electrolytes, some studies reveal that the full cell with Na_2_SO_4_ as electrolyte may show poor cyclability and NaCH_3_COONa (NaAc) electrolyte exhibits improved performance, for the NaMnO_2_ cathode suffered from dissolving excessively in the Na_2_SO_4_ electrolyte followed by rapid capacity loss of the full cell [159]. Although NaClO_4_ electrolyte showed even a little better performance than that of the Na_2_SO_4_ electrode, its explosiveness and oxidizing abilities may be concerns in the market [147]. In aqueous NaNO_3_ electrolyte, the NTP anode exhibits higher intercalation/deintercalation kinetics and reactions that are approximately twice as fast as in LiNO_3_ solution [57]; further, the NTP anode in Li^+^ ion aqueous electrolyte (1 M Li_2_SO_4_ solution) will suffer from a higher potential plateau together with a suppressed rate performance due to the thermodynamic limitations of the lithium insertion into the Na-containing structure and/or Na^+^–Li^+^ repulsive interactions [70]. Another study showed that the full cell (Na_2_FeP_2_O_7_//NTP) with a higher NaNO_3_ concentration exhibited a large irreversible capacity due to H_2_ gas evolution and corrosive side reactions [147]. NaOH aqueous electrolyte, however, showed poor performance in cycling stability, which may be due to the decreased stability of the electrode material at higher pH [47, 97, 141].

In aqueous electrolyte SIBs, the salt concentration of the electrolyte affects the ionic conductivity and the rate performance of batteries as well as the diffusion of the reactive species, which can cause self-discharge, particularly in high-mass-loading electrodes. Studies showed that with higher-molarity solutions of typical electrolytes, rate capability and electrode utilization increased significantly (the redox peaks are sharper and closer, which indicates faster kinetics, consistent with the ionic conductivity difference); e.g., by increasing the salt (NaClO_4_) concentration from 1 to 5 M, the capacity at 1.5 C increased by 38%, and the oxygen-related self-discharge phenomenon diminished, although measurable irreversible capacity loss still occurred with the lowest oxygen content, suggesting that self-discharge and capacity loss are not necessarily causally related [170]. Furthermore, the increase in electrolyte concentration extended the electrochemical window of ASIBs up to 2.8 V (with concentrated 17 mol kg^−1^ NaClO_4_ aqueous electrolyte compared to the value of only 1.9 V with diluted 1 mol kg^−1^, which widened the theoretical voltage restriction of 1.23 V due to practical overvoltage) and could produce a higher discharge plateau of 1.8 V in a full cell with the Prussian-blue-type cathode. Higher concentrations of electrolyte under a higher rate condition benefits the more stable performance in ASIB systems due to the reduced water content of the NTP anode and elution of the cathode by alkalization of the aqueous electrolyte [171]. For an extremely high concentration, superconcentrated “water-in-salt” electrolytes (WiSEs) with the decreased activity of water resulting from its coordination with concentrated salt ions (or much more intense cation–anion interaction and pronounced ion aggregation in Na-ion electrolytes revealed by Raman spectra together with molecular-scale simulations) can even noticeably suppress the electrochemical decomposition of aqueous electrolytes (yet a dense, stable, and repairable SEI simultaneously formed) and significantly enhance the long-term cycling stability (e.g., > 1200 cycles at 1 C with negligible capacity losses—0.006% per cycle; and showing an extraordinarily high CE > 99.2% at 0.2 C over 350 cycles) at both low and high rates [172, 173]. However, it needs to be mentioned that highly concentrated electrolyte can potentially raise challenging issues such as corrosion, especially at extreme electrochemical potentials [147, 170], and therefore there should be a balance between the electrolyte concentration and high performance. In addition, aqueous electrolytes with more extreme pH (i.e., pH > 13) or those exposed to higher temperatures (e.g., 70 °C) will induce significant structural degradation and precipitation of a secondary phase (i.e., via loss of phosphate to layered sodium titanate) [174]. An aqueous/non-aqueous hybrid electrolyte with an expanded electrochemical window of up to 2.8 V and high conductivity was also explored, which inherited the safety feature of aqueous electrolytes and the electrochemical stability of non-aqueous systems [175].

**Fig. 10:**
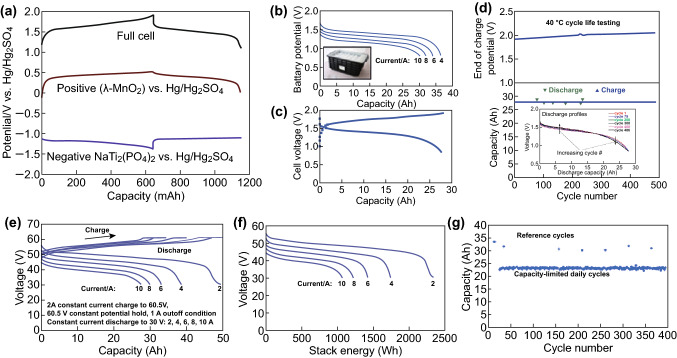
**a** Three electrode potential-limited constant-current charge/discharge data of a full cell containing the *λ*-MnO_2_ positive electrode and NTP negative electrode, showing the relative potential of the active materials during use. Cycle life data (**b**–**d**) (voltage profile shown in **b**; ± 4 A constant-current charge and discharge, with a capacity-limited charge and a voltage-limited discharge) from a large-format unit with 28 Ah capacity. These data were collected at a constant temperature of 40 °C, and this test is ongoing (nearly 800 cycles logged at this point). The maximum voltage reached upon full charge is just above 2 V under 4 A current, which is less than 1.8 V after IR correction. The test is ongoing and has been under way for 14 months. **e**, **f** Performance data for an eight-battery stack showing the delivered energy as a function of current as well as the long-term stability of the battery **g** [176]. Copyright 2014, Wiley–VCH Verlag

Although the neutral pH aqueous electrolyte SIBs with clean, non-flammable, fast internal ion transportation, and relatively lower manufacturing cost have shown considerable advantages, especially for large-scale energy storage applications (i.e., promising solutions for applications where constraints on energy density and weight are less rigid) compared with SIBs based on organic electrolytes, the stability window of water limits the voltage of an aqueous cell. Fortunately, researchers have found some solutions that enable the practical stability window of aqueous electrolytes to be widened beyond the theoretical limit via the kinetic effect, which enables the usage of materials whose operating potential exceeds the thermodynamic limit of pure water in an aqueous system [170, 176]. Despite the low cost and eco-friendly features of ASIBs that make them promising candidates for future energy storage systems, aqueous batteries are very much constrained by electrolyte degradation. Electrolyte additives (e.g., vinylene carbonate or other low-cost acetic additives) can further enhance the cycle stability of the full cell [177]. Date from the large-format energy storage device showed promising commercial application of ASIBs of cycle-stable high-voltage strings of cells (Fig. 10a–g) [176].

All-solid-state SIBs have attracted considerable attention for their safety and long-term durability [122–125]. Apart from the typical SIBs based on liquid electrolytes, solid-state rechargeable SIBs based on ceramic (e.g., Na-βʺ-Al_2_O_3_) electrolyte with high sodium-ion conductivity can demonstrate an extremely stable voltage plateau of ~ 2.1 V in the half-cell and an initial discharge capacity of 133 mAh g^−1^, although the cycling and rate performances may be improved via modification of interfacial incompatibility (or cell resistance and intrinsic polarization) compared to that of typical non-aqueous SIBs [126].

**Fig. 11:**
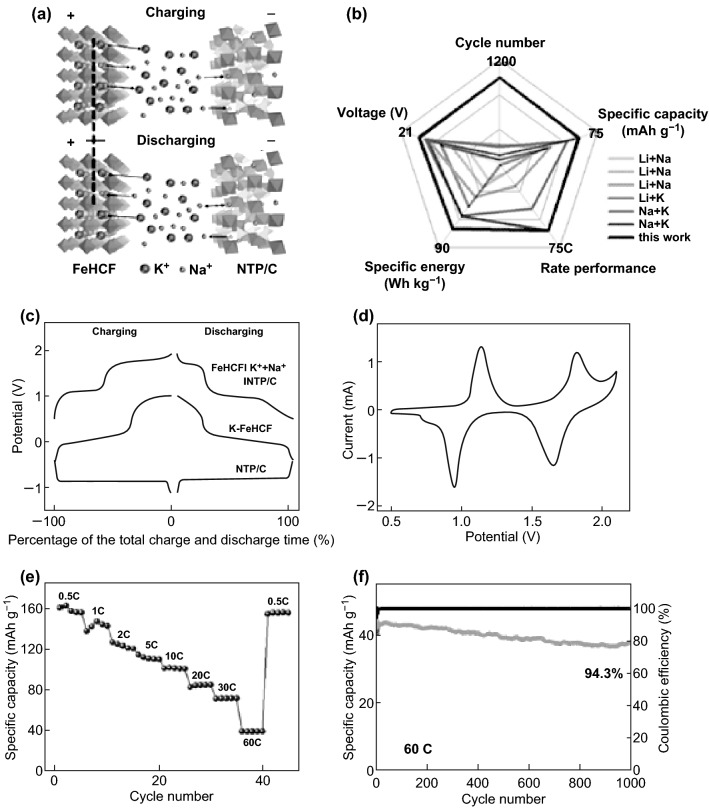
**a** Schematic of the K–Na hybrid aqueous battery (HAB). **b** Performance comparison between the HAB designed in this work and other previously reported mixed-ion aqueous batteries. **c** Galvanostatic profiles of the HAB along with the voltage profiles of their individual anode and cathode electrodes versus SCE at the rate of 1 C. **d** CV curves of the HAB at the scan rate of 1 mVs^−1^. **e** Rate performance of the HAB. **f** Long-term cycling stability of the HAB at the rate of 60 C [186]. Copyright 2018, Wiley-VCH Verlag

Aqueous rechargeable batteries (ARBs), or hybrid aqueous batteries (HABs), based on the migration of two or more types of shuttle ions, will not only enrich the battery families but will also make an operation voltage higher than 1.2 V more available for ARBs [176, 178–192]. For example, NTP–C//LMO (LiMn_2_O_4_) full cells with Li^+^/Na^+^-mixed electrolyte exhibited enhanced electrochemical performance and anode-dependent electrochemical behavior [178]. For the Li^+^/Na^+^-mixed electrolyte, both NTP and its counterpart, LiTi_2_(PO_4_)_3_, exhibited better rate performance due to the lower diffusion barrier in the NASICON structure compared to that of an electrolyte consisting of single Li^+^ ions; the Li or Na ions in the M1 sites of the NASICON materials will be replaced by the ions from the electrolytes with top priority for Na ions [70]. For HABs consisting of electrode materials with selective cation channels, the electrode applicability could increase and thus broaden the application of ARBs. Liu et al. constructed a high-voltage K-Na HAB based on a carbon-coated NTP (open holey nanocube structure) anode and a K_2_FeFe(CN) cathode to combine the respective advantages of each material and improve the rate performance and CE (Fig. 11a). Due to the unique cation selectivity of both electrode materials and the ultrafast ion conduction of NTP/C, the hybrid battery delivered a superior capacity of 160 mAh g^−1^ at 0.5 C rate (with an operating voltage ranging from 0.5 to 1.9 V and discharging plateaus at approximately 1.72 and 0.98 V), high rate capabilities (with excellent capacity recovery capability), and a record long-term capacity retention of 94.3% over 1000 cycles at even an ultrahigh rate of 60 C (Fig. 11c–f). Meanwhile, a high energy density of 69.6 Wh kg^−1^ calculated on the total mass of active electrode materials could be obtained, which is comparable to or even superior to that of current commercial aqueous batteries (Fig. 11b) [186]. Using this strategy for integrating different electrode materials with unique cation selectivity toward metal ions, a high-voltage rechargeable aqueous battery will be realized with a high capacity, remarkable energy density, and considerable capacity retention at a high rate.

### Aqueous Batteries with Desalination

**Fig. 12:**
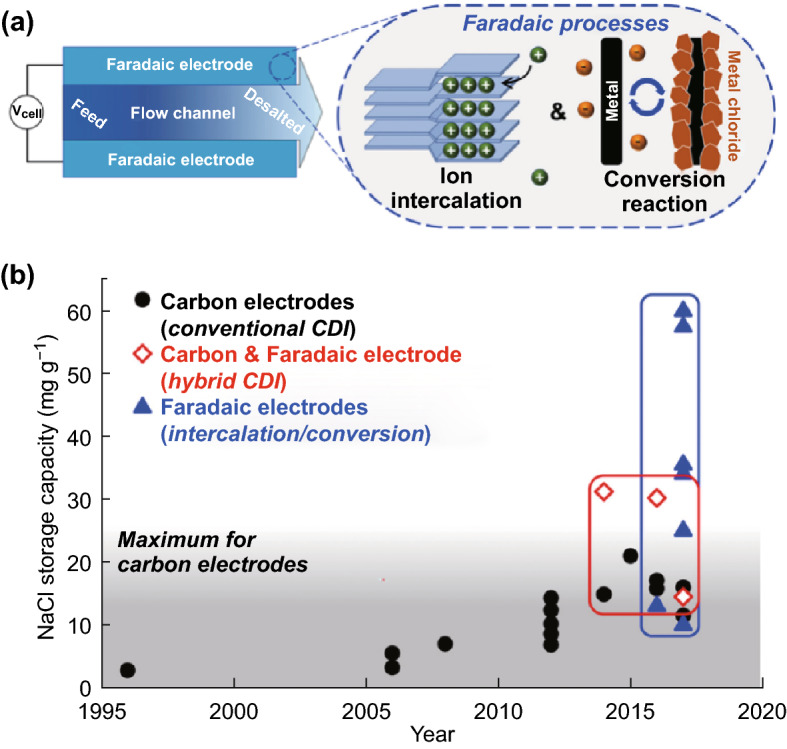
Concept and performance values of capacitive deionization (CDI) cells with capacitive and Faradaic electrodes. Historical evolution of the salt storage capacity of desalination cells achieved with either nanoporous carbon electrodes (black circles), Faradaic electrodes (blue triangles), or hybrid cells with one nanoporous carbon electrode and one Faradaic electrode (red diamonds), with all values reported per mass of both electrodes. Despite their recent introduction, cells with Faradaic electrodes have achieved significantly higher gravimetric salt storage capacities compared with cells with nanoporous carbon electrodes. Schematics contrast the salt storage mechanism of CDI cells leveraging nanoporous carbon (capacitive) electrodes and cells with Faradaic electrodes, such as layered intercalation electrodes or conversion electrodes [195]. Copyright 2017, Elsevier Inc. (Color figure online)

New desalination technologies with high ion removal capacity and low energy consumption are urgently needed to solve the worldwide water scarcity problem, and capacitive deionization (CDI) is now regarded as a competitive electrochemical means of saving energy and delivering clean water, i.e., water desalination using energy storage electrode materials with the benefit of energy recovery [193–198]. Faradaic cation insertion electrodes (e.g., NTP, NMO) have been widely used in electrochemical energy storage and more recently have been explored for selective electrochemical deionization applications due to the higher inherent ion selectivity and lower energy/material intensity arising from their specific crystalline structures and well-defined redox potentials [21, 193, 199–204]. Depending on the consistency with the lattice vacancy in the host’s crystal lattice, only smaller monovalent Li^+^ and Na^+^ ions may insert into the host compound’s crystal lattice, while the larger monovalent ions (e.g., K^+^) or divalent ions (e.g., Ca^2+^) are left behind in the solution, and the removal capacities could be 5–10 time higher than carbon materials with high preference to specific inserting ions (Fig. 12a, b) [195, 198, 199]. Taking the NTP/rGO composite as an example, in the novel hybrid electrochemical deionization (EDI) system, Na^+^ ions in the saline water will be intercalated into the NTP/rGO electrode via a chemical reaction, while Cl^−^ ions are physically adsorbed on the other AC electrode. The EDI system showed an ultrahigh desalination performance with an initial salt removal capacity of 140 mg g^−1^ at a current density of 100 mA g^−1^, and retaining 120 mg g^−1^ over 100 cycles. A particularly rapid desalination rate of 0.45 mg g^−1^ s^−1^ can be achieved at 1000 mA g^−1^ with a removal capacity of 27 mg g^−1^ [22]. Through further replacement of the AC electrode with an Ag nanoparticles/rGO composite electrode, a lower-energy-consumption dual-ion electrochemical deionization system was designed, where NTP/rGO served as a Na^+^ ion Faradaic electrode and Ag-NPs/rGO as a Cl^−^ ion Faradaic electrode. The estimated energy consumption can be as low as 0.254 Wh L^−1^ for the desalination of brackish water (2500 ppm) to drinkable water (500 ppm). However, because currently few materials can be chosen as an efficient anion Faradaic electrode, the development of cost-efficient battery materials will also accelerate desalination technology [204–207].

Further study showed that the ion transport from the bulk electrolyte to the electrode surface limited the rate of ion removal and the round-trip CE, and the presence of non-inserting ions in water would reduce the ionic flux and ion removal capacity due to the containment of interfacial transport following the accumulation effect [199]. Generally, optimizing the operating current density and cutoff voltage window (to reduce the parasitic water splitting reaction) as well as advective ion transport to the electrode surface will improve the ion removal performance in dilute aqueous systems [199]. The excellent performance of the EDI system based on NTP composites has made it a promising desalination technology and provides significant potential for direct seawater desalination in the future. By combining commercial photovoltaics, the goal of “renewables to usable electric energy and desalted water” can be achieved [208].

**Fig. 13:**
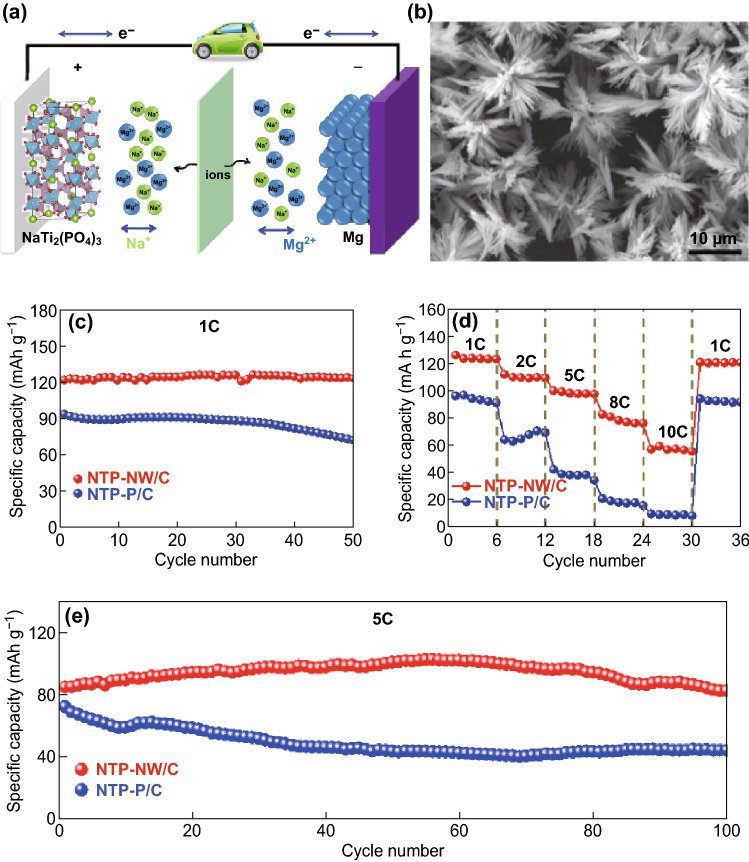
**a** Schematic illustration of the Mg–Na hybrid battery in this work. **b** FESEM image of NTP-NW/C. **c** Cycling performances of NTP-NW/C and NTP-P/C at 1 C. **d** Rate performances of NTP-NW/C and NTP-P/C. **e** Cycling performances of NTP-NW/C and NTP-P/C at 5 C [211]. Copyright 2018, Elsevier Ltd

Apart from the above-discussed SIBs, some other new types of energy storage systems such as Mg–Na hybrid ion batteries based on the NTP-based electrode have also been reported [185, 209, 210]. Mai’s group first reported the novel NTP nanowire clusters as a hybrid magnesium–sodium-ion battery’s cathode with the combined advantages of a fast alkali metal ions intercalation cathode and a dendrite-free Mg anode and exhibited good electrochemical performance with a high reversible capacity of 124 mAh g^−1^ at 1 C, considerable rate capability, and good cycling stability (Fig. 13c–e) [211]. As shown in Fig. 13a, b, this innovative device consists of an anode of Mg metal, a cathode of NTP nanowire clusters, and a dual salt electrolyte consisting of the common MIB electrolyte with a suitable Na salt added to it. Because of the open structure of the self-assembled NTP nanoarchitecture and the suitable potential of 1.7 V (vs. Mg/Mg^2+^) of NTP, NTP nanowire clusters can be potentially used as the cathode for magnesium–sodium hybrid ion batteries.

## Hybrid Capacitors

In the past few years, many articles on NHCs or hybrid sodium-ion capacitors (NICs, or SICs) have been published [37, 212–215]. Apart from the high-electrical-conductivity carbon-based materials and high-power-performance metal oxides such as TiO_2_, Nb_2_O_5_ and so on, NaTi_2_(PO_4_)_3_ with a NASICON structure features high ionic conductivity and structural stability with the excellent kinetics of sodium, and therefore will be a suitable material for sodium-ion hybrid capacitor applications [32, 212, 216, 217]. However, it has poor electron conductivity, and therefore many methods such as reducing the particle size, coating conductive materials, adopting suitable counter electrode materials, etc., have been used to rationally design NTP materials to solve these problems [218, 219].

Zhang and his coworkers first reported that a NaTi_2_(PO_4_)_3_/C composite synthesized by the ball milling method used as the anode of an aqueous sodium-ion hybrid supercapacitor with AC as the cathode could deliver a high energy density of 31.6 Wh kg^−1^. When tested at a current density of 200 mA g^−1^, the cell delivered an excellent cycle performance of less than 11.7% capacitance loss after 2000 cycles. This electrochemical performance is derived from the unique structure of the as-synthesized NaTi_2_(PO_4_)_3_/C composite. First, the NASICON structure of NaTi_2_(PO_4_)_3_ could promote rapid and easy migration of Na^+^ ions in the 3D open framework structure. On the other hand, the intimate mixing of acetylene black and sucrose with the precursor material through ball milling results in the formation of a uniform amorphous carbon layer of approximately 7 nm on the surface of the NTP particles so that the electron conductivity of the NaTi_2_(PO_4_)_3_/C composite could be significantly improved [218]. However, the as-synthesized NaTi_2_(PO_4_)_3_/C composite is in the range of 0.5–2 mm, which might be detrimental to the diffusion of ions and electrons in the electrode material. Roh et al. reported a NaTi_2_(PO_4_)_3_/rGO microsphere composite synthesized by a facile spray-drying method used as a high-rate insertion anode for sodium-ion capacitors. The spray-drying method produced a structure of NTP nanoparticles with sizes < 80 nm, which considerably reduced the diffusion length of the Na^+^ ion inside the material. Moreover, during the synthesis process, components of titanium were ionic species, which caused the chemical bonding of high-conductivity reduced graphene oxide (rGO) with NTP and finally significantly improved the electrical conductivity of the composite. When fabricated with an AC counter electrode to construct an NHC, a maximum energy density of 53 Wh kg^−1^ at a power density of 334 W kg^−1^ with good cycling stability was obtained [220].

Similar work has also been reported by Lee and his coworkers. They synthesized NTP nanoparticles grown on graphene nanosheets as an anode with the graphene nanosheets as a cathode in an organic electrolyte NHC. This new system features a high specific surface area and a high-conductivity nanosheet-like graphene cathode. Unlike the activated carbon electrode, which is porous with pores that are not conducive to electron transport, the surface of the 2D nanosheet can be easily contacted by ions in the electrolyte, thereby reducing the ion transport distance. This new system delivers a high energy density of ≈ 80 Wh kg^−1^ and a high specific power of 8 kW kg^−1^. An ultralow performance fading of ≈ 0.13% per 1000 cycle (90%–75,000 cycles) outperforms previously reported sodium-ion capacitors [32].

Electrospinning is a classic method of preparing a 3D network carbon nanofiber with uniform morphology and good electrical conductivity. Recently, Wei et al. reported that porous NTP/C nanofibers (NTP/CNFs) obtained via an electrospinning method’s anode of NHCs could deliver a maximum specific energy density of 56 Wh kg^−1^ at a specific power density of 174 W kg^−1^. At a current density of 1 A g^−1^, the specific capacitance remained at 91.4% after 500 cycles with nearly 100% CE. The electrospinning method uniformly dispersed the NTP nanoparticles with an average crystal size of ~ 15 nm in the carbon matrix. Characterizations suggested that NTP/CNFs have a typical porous structure with a high specific surface area that will facilitate electrolyte infiltration and finally produce high electrochemical performance [75].

**Fig. 14:**
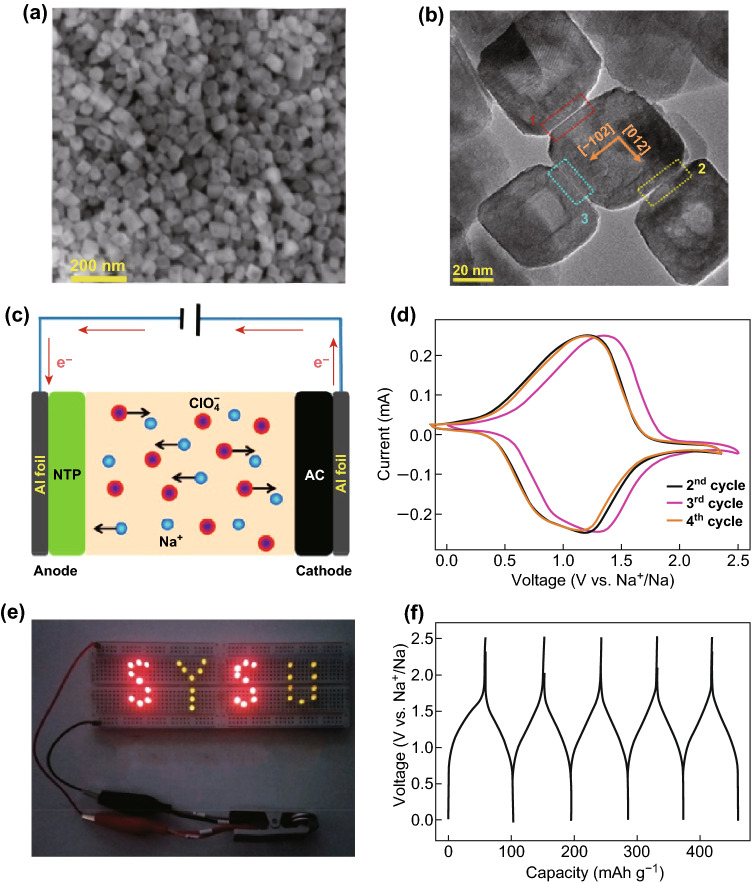
**a** SEM and **b** TEM images of the obtained mesoporous NTP nanocages. **c** Schematic illustration of the operating mechanism of an NHC. **d** Photograph of a logo consisting of 35 LEDs powered by two NHCs in series. **e**, **f** CV curves and charge–discharge profiles of the sodium-ion capacitor [221]. Copyright 2017, American Chemical Society

Recently, Wei et al. reported mesoporous NTP nanocages with iso-oriented tiny nanocrystals (Fig. 14a, b) synthesized by the solvothermal method used as the anode for an NHC. The full cell (with AC as the positive electrode, NTP nanocages as the anode, and 1 M NaClO_4_ in PC as the electrolyte) combines the advantages of the batteries and supercapacitors, i.e., relative high capacitance, outstanding rate performance, and long cycling stability (with obvious humps between 0.9–1.6 V in CV curves due to the rapid insertion/extraction of Na^+^ with the NTP electrode accompanied by the adsorption/desorption of NaClO_4_^−^ with the AC electrode, corresponding to the well-matched stable charge–discharge profiles) (Fig. 14c–f). It could deliver an energy density of 56 Wh kg^−1^ at a power density of 39 W kg^−1^ and excellent cycle performance without obvious capacity degradation after 20,000 cycles even at a high current rate of 5 A g^−1^. SEM and TEM images show that the products display a hollow structure with cube-like morphologies in the range 20–50 nm. The N_2_ sorption isotherm confirmed the porous nanostructures with a specific surface area of 67.4 m^2^ g^−1^; such a unique porous nanostructure results in more active sites, shorter ion transport routes, and finally promotes sodium-ion transport dynamics [221]. With the merits of impressive energy and power densities as well as cycling performance, the hybrid capacitor could be a promising device for high-efficiency energy storage systems.

**Fig. 15:**
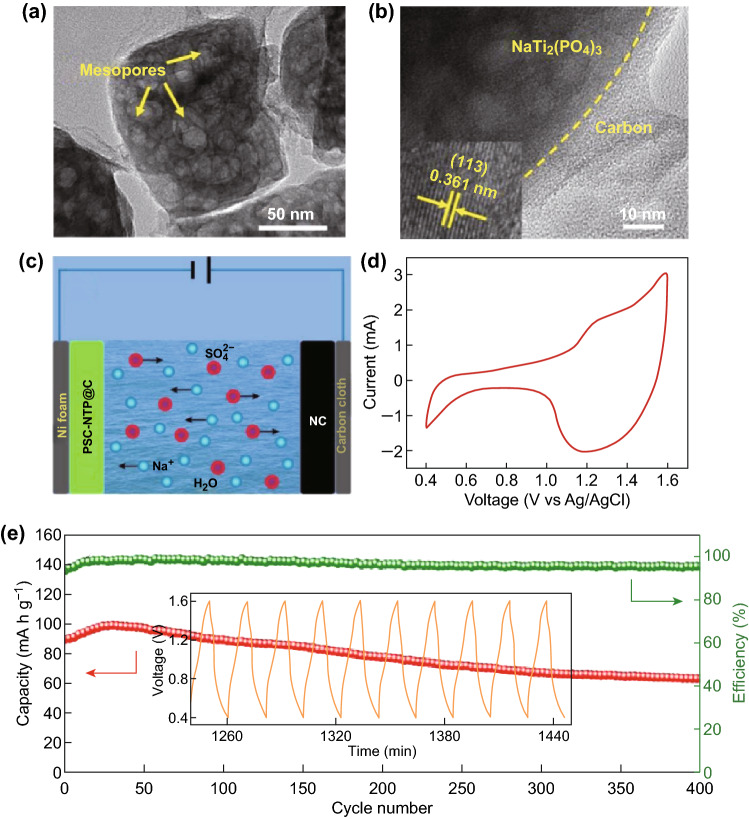
**a** TEM image reveals the porous nanostructure of single-crystal NaTi_2_(PO_4_)_3_ coated with amorphous carbon layer (PSC-NTP@C); **b** HRTEM image at the interface between an NTP and the carbon layer. **c** Schematic illustration of the PSC-NTP@C//NC Na-ion capacitor in liquid aqueous electrolyte; **d** CV curve of this Na-ion capacitor recorded at 3 mV s^−1^ within a cutoff voltage window of 0.4–1.6 V. **e** Cycle performance of the PSC-NTP@C//NC system at 0.5 A g^−1^ (the inset shows the voltage–time profile) [38]. Copyright 2018, Elsevier Ltd

After this work, Yang and his coworkers synthesized a porous single-crystal NTP by liquid transformation of ultrathin TiO_2_ nanosheets, followed by mixing with phenolic resin and calcining in an inert atmosphere to coat the conductive carbon sheath (denoted by PSC-NTP@C) (Fig. 15a, b). By comparing with partially crystallographic solid spheres that were NTP-fabricated via liquid transformation before annealing, the mesopores were determined to be derived from shrinking of amorphous nanodomains. The high crystallinity results in a more robust structure that enabled NTP to store sodium ions without causing large lattice stresses, finally helping improve cycle performance; at the same time, the thin amorphous carbon layer confirmed by TEM images could improve the efficiency of ion transfer in the electrode, resulting in excellent high rate performance, outstanding safety, and excellent flexibility [38]. When coupled with ZIF-8 derived N-doped porous carbon (NC) as the cathode material to assemble an aqueous Na-ion capacitor, the majority of the capacitance existed in the range 1.0–1.6 V for the PSC-NTP@C//NC capacitor, which showed a high initial discharge capacity of ~ 90 mAh g^−1^, good cycle stability, and high reversibility (Fig. 15c–e). It could further be fabricated to a flexible quasi-solid-state Na-ion capacitor with a sandwich structure, showing superior bendability for aqueous energy storage systems.

## Conclusion and Perspectives

In summary, the NASICON-type NTP-based electrode materials with exceptionally high ion conductivity and pronounced structural stability overcome the multiple kinetic problems of Na-ion systems, facilitating low-cost large-scale electrochemical energy storage with inhibited capacity decay, higher rate capacities, and CEs. When paired with existing competent cathodes, the class of NTP anode materials for SIBs shows advantages comparable with or superior to commercial high-power LIBs. Furthermore, among the many cutting-edge anode materials identified to deliver promising results, with some outperforming their lithium equivalents, NTP demonstrates a zero-strain insertion feature as well as a high ICE and a relatively high Na insertion/deinsertion, avoiding the formation of SEI and ensuring the safety of large-scale and high-power batteries [58]. The full volume change of the NTP anode with other paired cathodes is almost zero because they share approximately the same but inverse volume change during charging and discharging, which facilitates the realization of safe, long-term cyclability and flexible structure design for large-capacity batteries. On the basis of the above encouraging results, it is concluded that SIBs (including aqueous and non-aqueous), as well as NHCs, have shown significant potential for commercialization in the near future while there is plenty of room for the development of energy storage devices with higher energy densities and long-term lifespan. These areas should be the focus for future relevant research as follows:

1. Despite the fact that NTP has considerable potential for the development of high-performance SIBs, due to its relatively high voltage plateau as anodes, when used for a full cell, it will require cathodes with a high discharge potential to match and realize high power output. Thus, a higher technological requirement for the cathode materials should be proposed [45, 85, 107, 222]. From the viewpoint of practical battery applications, a high tap density is desirable for higher energy density in addition to high-power performance. In addition, the self-discharge rate has not been systematically evaluated, compared to the typical commercial LIBs with a value of 2% per month; the NTP-based SIBs and NHCs should be further investigated for practical application.
2. Although the ASIBs are more cost-effective and safer for large-scale energy storage, they often have a lower capacity and cycling life compared to that of organic SIBs. Because capacity fade in aqueous electrolytes remains poorly understood, further studies are needed, including a possible multi-step mechanism followed by a local pH change and alkaline oxidation of the carbon conductive additives [148].
3. The decay mechanism and stability of electrode materials in an aqueous electrolyte should be further studied and improved, although some efforts have shown promise by tailoring the electrolytes (including adjusting the pH values, locally generated destructive OH^−^ ions), nanocoating the electrode materials, or eliminating oxygen in the electrolytes to suppress capacity fading upon cycling. An appropriate potential window (cutoff voltage) and corresponding anode and cathode materials (with adjusted mass ratio) should be selected to avoid or suppress the highly irreversible capacity loss due to H_2_ and/or O_2_ evolution in the aqueous electrolytes.
4. For alternative electrolytes, the intrinsically safe organic phosphates for all-phosphate SIBs efficiently avoid firing as usually encountered in the carbonate electrolytes and severe side reactions such as hydrogen and oxygen evolution in aqueous electrolytes. The all-solid-state SIBs with safety, long-term operation capacity, and high-temperature performance advantages show considerable commercial potential. However, more related research on the interface contact is necessary, and the cycling performance of these batteries needs to be further improved for wide practical application.
5. The realization of full cell SIBs or hybrid Na-ion capacitors with high energy and long cycle life remains challenging. The controlled formation of an SEI on both anodes (especially for the hard-carbon-incorporated composites) and cathodes will be an effective way to achieve long-term stability for full cells. Pre-cycling (or pre-sodiation) of anodes and cathodes will lead to pre-formation of SEI, and hence mitigate the additional consumption of Na ions in full cells for higher ICE as well. With the improvement in aqueous electrolytes, including highly concentrated and even superconcentrated WiSEs or hybrid aqueous/non-aqueous electrolytes, the prospects are promising for large-scale and high-energy-density electrochemical energy storage with the advantages of low cost, eco-friendliness, and long lifespan.
6. Compared to conventional SIBs relayed on Cu/Al current collectors to support active materials and to serve as conductive pathways, free-standing or flexible electrodes (including graphene papers, graphene foams, and electrospun CNFs) without these metallic current collectors significantly reduce the weight and cost of batteries and have been an emerging demand for today’s battery development. However, more efforts are needed to develop better-performing free-standing electrode materials with a facile preparation route, low cost, and robust mechanical advantages for next-generation batteries.
7. Compared with traditional static capacitive deionization (CDI) using carbon electrodes, Faradaic capacitive (intercalation) electrodes including NTP can remove ion species with high efficiency (typically, an ultrahigh salt removal capacity of more than 100 mg g^−1^). These electrodes are promising for not only being a commercially viable alternative for treating water but also for saving energy. However, the stability of these electrodes (particularly associated with electrochemical leakage of metal ions) may be currently a major concern. Furthermore, it is necessary to develop robust and cost-effective intercalation cathodes and anodes to meet the critical requirement of capturing multiple cations and anions from real saline water or seawater.

## Electronic supplementary material

Below is the link to the electronic supplementary material.

Supplementary material 1 (PDF 437 kb)
