# Supplementary material for: NASICON-Structured NaTi2(PO4)3 for Sustainable Energy Storage
Source: Nanomicro Lett. 2019 May 25;11:44. doi: 10.1007/s40820-019-0273-1 (PMC7770786; doi:10.1007/s40820-019-0273-1)
Supplement: Supplementary file 1 — Supplementary material 1 (PDF 437 kb) [file 40820_2019_273_MOESM1_ESM.pdf]

Supporting Information for

NASICON-Structured $NaTi_2(PO_4)_3$ for Sustainable Energy Storage

Mingguang Wu[1], Wei Ni[2, 3, *], Jin Hu[1,*], Jianmin Ma[1, 4, *]

[1]School of Physics and Electronics, Hunan University, Changsha 410082, People's Republic of China

[2]Faculty of Technology, University of Oulu, Oulu 90014, Finland

[3]Panzhihua University, Panzhihua 617000, People's Republic of China

[4]Key Laboratory of Materials Processing and Mold (Zhengzhou University), Ministry of Education, Zhengzhou University, Zhengzhou 450002, People's Republic of China

*Corresponding authors. E-mail: wei.ni@oulu.fi (W. Ni); hujin@hnu.edu.cn (J. Hu); nanoelechem@hnu.edu.cn (J. Ma)

Table S1 Summary of the Na-storage performance of typical $NaTi_2(PO_4)_3$ in organic electrolytes. The theoretical capacity of $NaTi_2(PO_4)_3$ (NTP) as SIB anode is 133 mAh g^{-1} [S1]. EC: ethylene carbonate, DEC: diethyl carbonate, DMC: diethyl carbonate, PC: propylene carbonate; FEC: fluoroethylene carbonate. 1C = 133 mA g^{-1} [S2]. The value of rate C may vary in some references

Type of materials	Methods/Electrolytes	Current density (mA g^{-1})	Cycle number	Cut-off voltage (V)	Specific capacity (mAh g^{-1})	CE (%)	ICE (%)	Initial capacity Discharge/Charge	Rate capacity (Current density) (mAh g^{-1})	Mass loading (mg cm^{-2})	Year/Refs.
**Porous $NaTi_2(PO_4)_3$ nanocubes**	one-pot solvothermal<br>1 M $NaClO_4$ in EC/DMC (2% FEC)	10C	10000	1.5-2.8	87	99.96			110 (0.2C)<br>107 (0.5C)<br>106 (1C)<br>105 (2C)<br>104 (5C)	2.8-3.5 (1.6-2.1, active material)	2015/[S3]
**NTP-C (acetylene black) [NTP-C//$Na_2FeP_2O_7$]**	ball-milling, carbothermal treatment<br>1 M $NaClO_4$ in PC	2 mA cm^{-2}	30	0-1.4	[47]		[95]	[50/47]			2016/[S4]
**Carbon-coated $NaTi_2(PO_4)_3$ composite (NTP@C)**	evaporation, annealing<br>1 M $NaClO_4$ in EC/DMC	0.2C/5C	20	1.0-3.0	111/86			113@0.2C	109 (0.5C)<br>105 (1C)<br>99 (2C)<br>87 (5C)		2018/[S5]
**Porous $NaTi_2(PO_4)_3$ nanoparticles coated with thin carbon layer (mesoporous NTP@C)**	hydrothermal, PDA coating, annealing	1C/10C	500/2000	1.5-3.0	78/61		87.5	96/84@1C	109 (0.2C)<br>102 (0.5C)<br>98 (1C)<br>92 (2C)<br>84 (5C)<br>73 (10C)<br>55 (20C)		2018/[S6]
**$NaTi_2(PO_4)_3$/N-doped C composite**		10C (0.2C)	200 (30)	1.5-3.2	76 (126)			82@10C	124 (0.5C)<br>119 (1C)<br>113 (2C)<br>105 (4C)<br>95 (6C)		2018/[S7]
**$NaTi_2(PO_4)_3$/C porous plates**	solvothermal, in-situ carbon coating, calcination<br>1 M $NaClO_4$ in PC	10C (1/2/5C)	120	1.5-3.3	70 (105/100/82)	99 (~100 @1C/2C)		125@0.1C<br>110@1C<br>106@2C<br>95@5C<br>85@10C		2.3-2.9	2016/[S8]
**$NaTi_2(PO_4)_3$ nanoparticles embedded in interconnected nanocarbon networks (NTP/C)**	soft-template (CTAB), calcination<br>1 M $NaClO_4$ in EC/DMC	50C (1C/5C/10C/20C)	6000 (800/2000/3000/1000)	1.5-2.8	83 (119/~113/118/118)	~100 (~100 @5C)	~98 @1C	132@iC<br>126@5C<br>122@10C	132 (1C)<br>125 (5C)<br>124 (10C)<br>123 (20C)<br>120 (40C)<br>114 (60C)	~0.6	2016/[S9]

									109 (80C)<br>108 (100C)		
**Nanostructured $NaTi_2(PO_4)_3$/C**	pyro-synthesis, annealing<br>1 M $NaClO_4$ in EC/DEC (3% FEC)	5C<br>(0.5C)	250<br>(70)	0.01-2.8	~150<br>(179)	~100		316@0.5C	225 (0.2C)<br>181 (5C)		2016/[S10]
**NTP@C nanocomposite**	sol-gel, grinding, sintering<br>1 M $NaClO_4$ in EC/PC	20C	10000	0.01-3.0	56		48	455	170 (1C)<br>115 (10C)<br>66 (50C)<br>50 (100C)	1.6-2.4	2016/[S11]
**NTP/C<br>[full cell with ternary-metal PBAs cathode]**	hydrothermal, heating (glucose-derived C)<br>1 M $NaClO_4$ in EC/DEC (2% FEC)	[150]	[300]	[0.5-2.2]	[~110]	[~100]	[78.2]	[161/126@15 mA g^{-1}]<br>[based on PBAs]	[90@750<br>60@1500 mA g^{-1}]		2018/[S12]
**NTP/C nanocomposites**<br>(NPs size 20-40 nm, C shell thickness ~4nm, C content 3.82%)<br>[full cell NTP/C-U//$Na_3V_2(PO_4)_2F_3$]	solvothermal, in-situ carbon coating (calcination)<br>1 M $NaClO_4$ in DMC/EC/EMC (5% FEC)	1C/3C<br>[1C]	1500<br>[200]	1.5-3.0	97/~97<br>[~92]			107	109@0.5C<br>92@20C<br>86@30C<br>66@50C<br>[111@0.5C<br>93@10C<br>69@20C]	~1.5	2018/[S13]
**$NaTi_2(PO_4)_3$ nanoparticles**	polyol-assisted pyro-synthesis, annealing<br>1 M $NaClO_4$ in EC/DEC (3% FEC)	0.1C	30	1.2-2.8	208				110 (0.2C)<br>98 (0.5C)<br>86 (1C)<br>79 (2C)<br>65 (5C)<br>56 (10C)<br>42 (20C)		2016/[S14]
**3D $NaTi_2(PO_4)_3$@C microspheres**	spray-drying, calcination<br>1 M $NaClO_4$ in EC/DMC	10C<br>(0.1C)	500<br>(30)	1.5-2.8	97<br>(124)			127@0.1C	124 (0.5C)<br>120 (1C)<br>116 (2C)<br>107 (5C)	2.6	2018/[S15]
**Open holey-structured $NaTi_2(PO_4)_3$/C nanocomposite**	solvothermal, annealing<br>1 M $NaClO_4$ in EC/DMC	50C<br>(0.5C)	10000<br>(200)	1.5-3.0	103<br>(123)			129@0.5C	124 (0.5C)<br>120 (50C)		2018/[S16]
**Nanosized porous carbon-coated $NaTi_2(PO_4)_3$ particles**	hydrothermal, annealing	10C<br>(0.5C)	1000<br>(350)	1.5-3.0	106<br>(108)		95.2	/115@0.1C<br>/115@0.5C	114 (0.5/1/2C)<br>113 (5/10C)<br>112 (20C)<br>111@30C	1.35	2018/[S17]

**(mesoporous NTP@C nanocubes)**	1 M $NaClO_4$ in EC/DMC (1% FEC)								[-20ºC: 98@10C<br>61@20C]		
**$NaTi_2(PO_4)_3$/C composite nanofibers**	electrospinning, calcination<br>1 M $NaClO_4$ in EC/PC (5% FEC)	5C	500	15-3.3	97	~100		129@0.1C<br>105@5C	130 (0.1C)<br>123 (0.2C)<br>122 (0.5C)<br>119 (1C)<br>114 (2C)<br>103 (5C)<br>87 (10C)<br>63 (20C)	~1.7	2017/[S18]
**Porous $NaTi_2(PO_4)_3$/C hierarchical nanofibers [full cell NTP/CNFs//NiHCF]**	electrospinning, annealing<br>1 M $NaClO_4$ in EC/DEC (2% FEC)	2C	700	1.5-3.0	~110	~100			120 (0.2C)<br>118 (1C/2C)<br>111 (5C)<br>98 (10C)<br>71 (20C)		2018/[S19]
**Ultrafine $NaTi_2(PO_4)_3$ NPs encapsulated in N-doped CNFs (NTP-NCNFs)**	electrospinning, calcination<br>1 M $NaClO_4$ in EC/PC	10C	2000/20000	0-3.0	121/105				176 (0.1C)<br>163 (0.2C)<br>149 (0.5C)<br>138 (1C)<br>127 (2C)<br>110 (5C)<br>95 (10C)<br>71 (20C)	0.64	2018/[S20]
**$NaTi_2(PO_4)_3$/C nanocomposite (NPT/C-CNTs)**	solvothermal, hydrothermal, annealing<br>1 M $NaClO_4$ in EC/PC	1C/50C	200/1000	1.5-3.0	109/94	~100@50C		112@1C<br>96@50C	117 (1C)<br>113 (10C)<br>103 (50C)<br>[0.5C, 118@0ºC, 117@-10ºC, 113@-20ºC]<br>[65 (10C) at -20ºC]	~2	2016/[S21]
**Hierarchical porous nanocomposite of MWCNT-threaded mesoporous $NaTi_2(PO_4)_3$ nanocrystals**	hydrothermal, calcination, hetero-assembly, freeze-drying<br>1 M $NaClO_4$ in EC/PC	1C/10C	200/2000	1.5-3.0	106/74	99	99	121/120@1C	102 (0.5C)<br>95 (2C)<br>90 (5C)<br>84 (20C)<br>82 (30C)<br>74 (50C)	0.9-1.76	2016/[S22]
**3D $NaTi_2(PO_4)_3$@CNT microspheres**	spray-drying, calcination<br>1 M $NaClO_4$ in EC/DEC	10C	20	1.5-3.0	88			127@0.1C	125 (0.2C)<br>123 (0.5C)<br>120 (1C)<br>116 (2C)<br>111 (4C)<br>105 (6C)	1.9	2017/[S23]

									97 (8C)<br>88 (10C)		
**Porous $NaTi_2(PO_4)_3$ nanocubes anchored on porous carbon nanosheets**	calcination<br>1 M $NaClO_4$ in EC/DEC (5% FEC)	100/4000	100/2000	0.01-3.0	172/98		46.8	485/227	280 (20)<br>164 (200)		2018/[S24]
**Mesoporous $NaTi_2(PO_4)_3$/CMK-3 nanohybrid**	solvothermal, calcination<br>1 M $NaClO_4$ in EC/PC	0.5C	1000	1.0-3.0	63	~100	98	102/100@0.2C	101 (0.2C)<br>76 (0.5C)<br>58 (1C)<br>39 (2C)	~2	2014/[S25]
**1D mesoporous $NaTi_2(PO_4)_3$/carbon nanofiber**	electrospinning, two-step calcination (Ar, air)<br>1 M $NaClO_4$ in EC/DEC	0.5C/10C	20	1.5-3.2	124/93		99.2@0.2C	127/126@0.2C	127 (0.2C)<br>124 (0.5C)<br>121 (1C)<br>116 (2C)<br>105 (5C)<br>93 (10C)		2018/[S26]
**Porous $NaTi_2(PO_4)_3$@C nanocubes**	hydrothermal, annealing<br>1 M $NaClO_4$ in EC/DMC (5% FEC)	100/1000	100/1000	0.01-3.0	201/140				225 (50)<br>203 (100)<br>190 (200)<br>166 (1000)<br>158 (2000)<br>149 (4000)<br>135 (8000)		2018/[S27]
**Carbon-coated NTP in porous carbon matrix (double carbon coating, NTP@C@PC)**	soft-chemical method, calcination<br>1 M $NaClO_4$ in EC/DMC	1C/5C/10C/20C	1000/5000/6000/9000	1.5-2.8	113/103/76/70	~100@20C	95@1C	133@0.5C<br>127@1C<br>110@10C	121 (1C)<br>118 (2C)<br>116 (5C)<br>112 (10C)<br>104 (20C)<br>94 (30C)<br>64 (50C)		2015/[S28]
**Rutile TiO_2 and carbon coated $NaTi_2(PO_4)_3$ nanocubes (C/NTP-RT)**	hydrothermal, vaporization, annealing<br>1 M $NaClO_4$ in EC/DMC	5C/10C	2000/10000	1.2-2.8	78/72	100			131 (0.1C)<br>113 (0.2C)<br>96 (0.5C)<br>92 (1C)<br>89 (2C)<br>86 (5C)<br>84 (10C)<br>77 (20C)	2-3	2015/[S29]
**Carbon-coated hierarchical $NaTi_2(PO_4)_3$ mesoporous mciroflowers**	solvothermal, annealing<br>1 M $NaClO_4$ in EC/DEC (5% FEC)	20C<br>(1/5/10C)	10000<br>(200/500/3000)	1.5-3.0	85<br>(117/110/101)	~100@10C		125@1C<br>116@5C<br>114@10C	124 (1C)<br>120 (2C)<br>115 (5C)<br>111 (10C)<br>107 (20C)	~1.8	2016/[S30]

									100 (50C)<br>95 (100C)		
**NTP/graphene composite**	sol-gel, post heat-treatment<br>1 M $NaClO_4$ in EC/DEC	100	700	1.5-3.8	102			121		2-4	2014/[S31]
**NTP/rGO nanocomposite (nanocrystalline, ~60 nm)**	Pechini method, calcination<br>1 M $NaClO_4$ in PC (5% FEC)	(5 mV s^{-1})	1000	1.5-3.0	82	>99				0.5-1.0	2017/[S32]
**NTP@C nanoparticles embedded in 2D S-doped graphene sheets**	sol-gel, sintering<br>1 M $NaClO_4$ in EC/PC	10C/20C<br>(0.1C)	100/500<br>(40)	1.5-3.0	113/96<br>(129)			102@20C	130 (0.1C)<br>129 (0.5C)<br>127 (1C)<br>125 (2C)<br>120 (5C)	~2.8	2018/[S33]
**Porous $NaTi_2(PO_4)_3$ nanoparticles@3D graphene networks**	hydrothermal, annealing<br>1 M $NaClO_4$ in EC/DEC (5% FEC)	1C/10C	200/1000	1.5-3.0	101/77	98/99	79/90	138/109@1C<br>107/96@10C	117 (0.5C)<br>112 (1C)<br>105 (5C)<br>96 (10C)<br>85 (20C)<br>67 (50C)	~1.5	2015/[S1]
**NTP/rGO nanocomposite**	microwave-assisted solvothermal, freeze-drying, calcination<br>1 M $NaClO_4$ in EC/DEC	10C	1000<br>(640)	1.5-3.0	~111<br>(120 at 55 °C)	99.8		129@0.1C<br>(123@10C, 55 °C)	94 (50C)	2-3	2016/[S34]
**3D $NaTi_2(PO_4)_3$@graphene (NTP@rGO) microspheres** ***[full cell NTP@rGO//$Na_3V_2(PO_4)_3$/C]***	spray-drying, calcination<br>1 M $NaClO_4$ in EC/DEC	20C<br>[10C]	1000<br>[1000]	1.4-3.0<br>[0.4-2.0, full cell]	~80<br>[~81]	>99.5	98	130/128@0.2C<br>[132/128@0.1C]	130 (0.1C)<br>130 (1C)<br>124 (10C)<br>122 (20C)<br>103 (50C)<br>75 (100C)<br>38 (200C)<br>[88@50C]	1.5	2016/[S2]
**$NaTi_2(PO_4)_3$/rGO composite (NPs: 20-30 nm)**	polyol-assisted pyro-synthesis, calcination<br>1 M $NaClO_4$ in PC	20C	1000	1.0-3.0	62	100		132@0.07C<br>91@20C	100 (2.3C)<br>95 (9.2C)<br>78 (36.8C)		2016/[S35]
**$NaTi_2(PO_4)_3$@N-rGO composite**	calcination<br>1 M $NaClO_4$ in EC/DEC	20C	200	1.5-3.0	74			129@0.1C<br>84@20C	126 (0.5C)<br>123 (1C)<br>117 (2C)<br>108 (5C)		2017/[S36]

									96 (10C)		
**Hollow $NaTi_2(PO_4)_3$ nanocubes/rGO**	hydrothermal, calcination<br>1 M $NaClO_4$ in EC/DEC	3/20C<br>(1/3/5/10C)	500<br>(100)	1.2-2.8	103/60<br>(124/118/104/100)	99.8		128@1C	128 (0.1C)<br>125 (1C)<br>118 (3C)<br>116 (5C)<br>106 (10C)<br>90 (20C)<br>63 (50C)	1.5-1.8	2017/[S37]
**$NaTi_2(PO_4)_3$/rGO microspheres<br>[full cell with cathode $Na_3V_2(PO_4)_3$/rGO]**	spray-drying, annealing<br>0.8 M $NaClO_4$ in TMP (trimethyl phosphate) (10% FEC)	0.5C<br>[10C]	1000<br>[1000]	1.4-3.0<br>[0.4-2.0 full cell]	91<br>[56]	99.5<br>[99.7]	[87.7]	[104@0.2C]	128 (0.2C)<br>123 (0.5C)<br>118 (1C)<br>112 (2C)<br>104 (5C)<br>96 (10C)<br>87 (20C)<br>81 (30C)<br>[100 (0.5C)<br>89 (1C)<br>82 (2C)<br>75 (5C)<br>61 (10C)<br>38 (20C)]		2017/[S38]
**NTP@C nanocrystals/graphene sheets<br>(rGO-NTP@C)**	hydrothermal, calcination<br>1 M $NaClO_4$ in EC/DEC	0.1C	40	1.5-3.0	127			129@0.1C	125 (0.5)<br>120 (1C)<br>112 (2C)<br>99 (5C)<br>81 (10C)		2017/[S39]
**MXene@NTP-C nanohybrid**	solvothermal, calcination (together with phenolic resin)<br>1 M $NaClO_4$ in EC/DEC (5% FEC)	100<br>(1000/5000)	100<br>(2k/10k)	0.1-3.0	197<br>(143/109)	~100			208 (100)<br>198 (200)<br>182 (500)<br>166 (1000)<br>142 (2000)<br>113 (5000)<br>102 (10000)	0.8-1.0	2018/[S40]
**Hierarchical layered NTP/Mxene**	solvothermal, calcination<br>1 M $NaClO_4$ in EC/DEC	200/2000	500/2000	0.01-3.0	121/~62		76.3	198/151	145 (200)<br>67 (2000)	~1.0	2019/[S41]
**Flexible and binder-free $NaTi_2(PO_4)_3$/graphene film electrode<br>[full cell with cathode $Na_{0.44}MnO_2$]**	hydrothermal, annealing, vacuum drying (glass culture dish)	100/500	100/1000	1.0-3.0<br>[2.0-4.0]	121/~90		86<br>[82.3 % ful cell]	137/118@100 mA g^{-1}	137 (100)<br>108 (300)<br>99 (500)<br>93 (1000)		2018/[S42]

	1 M $NaClO_4$ in EC/DMC (5% FEC)										
**Free-standing 3D mesoporous NTP/rGO nanocomposite [full cell with cathode $Na_3V_2(PO_4)_3$]**	electrostatic self-assembly, freeze-drying, mechanical pressing, thermal treatment<br>1 M $NaClO_4$ in EC/PC	1C/5C<br>[1C]	200/1000<br>[100]	1.5-3.0<br>[0.4-2.0]	101/~68<br>[58]	99@5C<br>[99@1C]	98.8	114/113@1C<br>[59@1C]	122 (0.5C)<br>114 (1C)<br>106 (2C)<br>94 (5C)<br>85 (10C)<br>76 (20C)<br>68 (30C)<br>52 (50C)<br>[58@1C]		2018/[S43]
NTP/C composite (PVP derived C) **[full cell $NaTi_2(PO_4)_3$//$Na_3V_2(PO_4)_3$]**	sol-gel, sintering<br>1 M $NaPF_6$ in PC (2% FEC)	50C<br>[20C]	1000<br>[5000]	1.4-3.0<br>[0.5-1.5]	~82<br>[80]	~100<br>[~100]	98.3<br>[95]	102/100@0.5C<br>[107@0.5C]<br>[based on NVP; Cu current collector]	[107@0.5C<br>99@5C<br>85@20C]	3.5	2018/[S44]
CNTs-NTP@C particles composite	sol-gel, annealing<br>1 M $NaClO_4$ in EC/PC	2C	200		113		97.3	128/125@0.2C	125 (0.5C)<br>122 (1C)<br>116 (2C)<br>106 (5C)<br>94 (10C)	2.7	2018/[S45]
**Self-supported mesoporous NTP nanocrystals/MWCNTs film**	electrostatic assembling, vacuum filtration, annealing<br>1 M $NaClO_4$ in EC/PC	1C/10C	200/3000	1.5-3.0	123/100	99	99	141/140	133 (1C)<br>127 (2C)<br>118 (5C)<br>105 (10C)<br>91 (20C)<br>81 (30C)<br>64 (50C)		2018/[S46]
**Flexible mesoporous NTP/rGO-CNTs**	solvothermal, hetero-assembly, vacuum filtration, annealing<br>1 M $NaClO_4$ in EC/PC	1C<br>(10C)	200<br>(5000)	1.5-3.0	126<br>(88)	~99<br>(98)	90	146/132	132 (0.5C)<br>128 (1C)<br>123 (2C)<br>116 (5C)<br>107 (10C)<br>90 (20C)<br>73 (30C)		2017/[S47]
**NTP powder**	ceramic electrolyte	100	50	1.5-2.5	~100	100	91	133/121	130 (0.1C)<br>110 (0.2C)<br>60 (0.5C)	0.4	2016/[S48]
**All-solid-state SIBs (NTP, carbon black)**	ceramic NASICON electrolyte (performance at 65 ºC)	0.2C	70	1.5-2.8	102	99.7			110 (0.2C)<br>94 (0.5C)<br>75 (1C)	~2	2017/[S49]

Table S2 Summary of the Na-storage performance of typical $NaTi_2(PO_4)_3$ (NTP) in aqueous electrolytes

Type of materials	Methods	Current density ($mA\ g^{-1}$)	Cycle number	Cut-off voltage (V)	Specific capacity ($mAh\ g^{-1}$)	CE (%)	ICE (%)	Initial capacity Discharge/Charge	Rate capacity/ Current density ($mAh\ g^{-1}$)	Mass loading ($mg\ cm^{-2}$)	Year/Refs.
**$NaTi_2(PO_4)_3$ powder [NTP//Zn] [Ag/AgCl reference electrode]**	Pechini method 1 M Na_2SO_4	2.0 $mA\ cm^{-2}$	30	-0.4 to -0.9	~70	~100		123			2011/[S50]
**NTP/C [NTP/graphite] (Ag/AgCl reference electrode)**	Pechini method, pyrolysis (carbon source: citric acid) [ball-milling, annealing] 1 M Na_2SO_4	2C	50	-0.9 to -0.3	117 [80]			129@2C [118@2C]	128 (2C) 66 (20C) [100 (2C) 34 (20C)]		2016/[S51]
**NTP@C (Ag/AgCl reference electrode)**	sol-gel, calcination, thermal decomposition vapor deposition (TVD), heat-treatment 1 M Na_2SO_4 {1 M Li_2SO_4/Na_2SO_4}	200	50		~80 {~71}	~98				~3	2016/[S52]
**NTP/C-U nanocomposites** (NPs size 20-40 nm, C shell thickness ~4nm, C content 3.82%) [full cell NTP/C-U//$Na_4Fe(CN)_6$]	solvothermal (urea), in-situ carbon coating (calcination) 1 M Na_2SO_4	1C [1C]	300 [500]	[0.0-2.0]	79 [~71]	>98		84	[84@0.5C 70@20C]	2-3	2018/[S13]
**Carbon-coated NTP nanoparticles** (~100 nm) **[NTP@C//$Na_{0.44}MnO_2$]**	hydrothermal, calcination 1 M Na_2SO_4	1C	300	[0.7-1.3]	~74	[>99. 5%@ 1C]	[~92@0.2C]	[131/121@0.2C] [0.1-1.3 V]	[121 (0.2C) 114 (0.5C) 103 (2C) 68 (5C)]	42/60	2016/[S53]
**Polypyrrole coated NTP particles (NTP@PPy)**	solution polymerization 1 M Na_2SO_4	0.2C	50		39		65	104/68			2015/[S54]
**NTP@C/Ag particles (C/Ag coating layer)**	sol-gel, grinding, calcination, sintering	2C/5C	100/400		108/70	>95		127@2C 98@5C	128 (1C) 111 (2C) 85 (5C) 65 (10C)		2018/[S55]

	1 M Na_2SO_4										
**NTP/C<br>(carbon black, expanded graphite)<br>[NTP/C//activated carbon]<br>(Ag/AgCl reference electrode)**	solid-state method (ball-milling, sintering)<br>1 M Na_2SO_4	2C<br>[2C]	100<br>[500]		78<br>[62]	~100<br>[~100<br>]	89@1C	110/98@1C	98 (1C)<br>86 (2C)<br>67 (5C)<br>52 (10C)<br>[100 (1C)<br>90 (2C)<br>67 (5C)<br>55 (10C)]	~5	2018/[S56]
**NTP/CNTs-graphite<br>(Hg/Hg2SO4 reference electrode)<br>[full cell NTP/$Na_{0.44}MnO_2$]**	ball-milling, sintering<br>1 M Na_2SO_4	1C	100	0.1-1.4	82	>99.7		130@0.1C	75 (2C)	6.4	2014/[S57]
**NTP-C// $Na_{0.44}MnO_2$<br>[Hg/Hg2SO4 reference electrode]**	rapid microwave-assisted method, ball milling (graphite)<br>1 M Na_2SO_4	15.7	20	-1.8 to -0.5 V<br>[1.25-0.6]	56			72<br>[85]		30	2013/[S58]
**Wafer-like 3D porous NTP/C composite<br>(Ag/AgCl reference electrode)<br>[full cell NTP/$Na_{0.44}MnO_2$]**	self-assembly synthesis, annealing<br>1 M Na_2SO_4	2C	300		92	~100	75@2C	~130/119@1C<br>114@2C	63 (50C)<br>[114@1C]		2015/[S59]
**Frogspawn-like hierarchical porous NTP/C array<br>(core-shell structure)**	template (hollow carbon sphere), impregnation, annealing	1C/20C	400/2000		~113/~87	~100		127@1C		2-3	2015/[S60]
**NTP/graphene nanocomposite<br>[calomel reference electrode (SCE)]**	solvothermal, calcination<br>1 M Na_2SO_4	2C	100	-1.0 to -0.5	100	~90		104	110 (2C)<br>85 (5C)<br>65 (10C)<br>40 (20C)		2014/[S61]
**NTP/graphene composite<br>(Ag/AgCl reference electrode)**	sol-gel, post heat-treatment<br>1 M Na_2SO_4	5C/10C/20C	400/100/<br>2000	-1.0 to -0.5	70/60/46				129 (1C)<br>125 (2C)<br>100 (5C)<br>88 (10C)<br>64 (20C)<br>56 (40C)	2-4	2014/[S31]
**$NaTi_2(PO_4)_3$//$Na_{0.44}MnO_2$<br>(NTP–C nanoparticles)<br>[full cell]**	ball-milling, calcination	25C	450	0.5-1.4	~55			~100@9C	~105@3C<br>~100@15C<br>(0.1-1.4 V)	up to 17.6/43.7	2013/[S62]

	(pyrolysis,<br>glucose)<br>1 M Na_2SO_4 in DI water										
**NaTi2(PO4)3//Na3V2(PO4)3 (carbon-coated NTP//NVP)**	solid state method (ball-milling, sintering, heat treatment)<br>1 M Na_2SO_4	10 A g^{-1}	50	0.5-1.6	~25		76	71@2 A g^{-1}<br>[0.5-1.5 V]	[71@5C<br>58@10C]		2016/[S63]
**TiN modified $NaTi_2(PO_4)_3$ particles (NTP@TiN)**	solvothermal, calcination (NH_3; nitriding)<br>1 M Na_2SO_4	2C	100		92	89	74	132		5	2018/[S64]
**NTP-C particles<br>$NaTi_2(PO_4)_3$//$Na_2NiFe(CN)_6$<br>[full cell NTP//Na_2PB]**	stoichiometric, solid-state reaction<br>1 M Na_2SO_4	5C<br>[5C]	100<br>[250]	 [1.6-0.2]	95<br>[79]	~100	85	101@5C		~10	2013/[S65]
**NTP/C NPs composite<br>$NaTi_2(PO_4)_3$//$NaMnO_2$<br>[full cell]**	 2 M CH_3COONa aqueous solution	5C<br>[5C]	500<br>[500]	 [1.8-0.5]	86<br>[28]	~100		127@1C<br>117@5C<br>[33@1C<br>23@10C<br>20@20C]	100 (20C)	2-2.5	2015/[S66]
**NTP-C particles<br>$NaTi_2(PO_4)_3$//$Na_2CuFe(CN)_6$<br>[full cell NTP//NaCuHCF]**	stoichiometric, solid-state reaction<br>1 M Na_2SO_4	[2C/10C]	[100/1000]	[1.8-0.0]	[97/74]	[75.6]		[104@2C]	[104 (2C)<br>93 (5C)<br>86 (10C)<br>83 (20C)<br>70 (50C)<br>60 (80C)<br>50 (100C)]	~10	2014/[S67]
**NTP/MWCNTs composite<br>NTP/MWCNTs//$Na_{0.44}MnO_2$**	solvothermal,<br>1 M Na_2SO_4	2C<br>[2C0]	200<br>[10/60/240]	 [1.4-0.2]	92<br>[128/60/50]		84	122/103	120 (2C)<br>90 (5C)<br>80 (10C)<br>60 (20C)<br>[68 (5C)<br>45 (10C)<br>23 (20C)]		2014/[S68]
**NTP-C (acetylene black)<br>[NTP-C//$Na_2FeP_2O_7$]**	ball-milling, carbothermal treatment	2 mA cm^{-2}	30	1.4-0	[38<br>3<br>42]		[95 (2M Na_2SO_4)<br>82 (4 M $NaNO_3$)<br>96 (4M $NaClO_4$)<br>at 2 mA cm^{-2}]				2016/[S4]

Supplementary References

[S1] C. Wu, P. Kopold, Y.-L. Ding, P.A. van Aken, J. Maier, Y. Yu, Synthesizing porous $NaTi_2(PO_4)_3$ nanoparticles embedded in 3D graphene networks for high-rate and long cycle-life sodium electrodes. ACS Nano **9**, 6610-6618 (2015). https://doi.org/10.1021/acsnano.5b02787

[S2] Y. Fang, L. Xiao, J. Qian, Y. Cao, X. Ai, Y. Huang, H. Yang, 3D graphene decorated $NaTi_2(PO_4)_3$ microspheres as a superior high-rate and ultracycle-stable anode material for sodium ion batteries. Adv. Energy Mater. **6**, 1502197 (2016). https://doi.org/10.1002/aenm.201502197

[S3] G. Yang, H. Song, M. Wu, C. Wang, Porous $NaTi_2(PO_4)_3$ nanocubes: a high-rate nonaqueous sodium anode material with more than 10 000 cycle life. J. Mater. Chem. A **3**, 18718-18726 (2015). https://doi.org/10.1039/C5TA04491J

[S4] K. Nakamoto, Y. Kano, A. Kitajou, S. Okada, Electrolyte dependence of the performance of a $Na_2FeP_2O_7$//$NaTi_2(PO_4)_3$ rechargeable aqueous sodium-ion battery. J. Power Sources **327**, 327-332 (2016). https://doi.org/10.1016/j.jpowsour.2016.07.052

[S5] X. Yang, K. Wang, X. Wang, G. Chang, S. Sun, Carbon-coated $NaTi_2(PO_4)_3$ composite: A promising anode material for sodium-ion batteries with superior Na-storage performance. Solid State Ionics **314**, 61-65(2018). https://doi.org/10.1016/j.ssi.2017.11.016

[S6] D. Cai, B. Qu, H. Zhan, Porous $NaTi_2(PO_4)_3$ nanoparticles coated with a thin carbon layer for sodium-ion batteries with enhanced rate and cycling performance. Mater. Lett. **218**, 14-17 (2018). https://doi.org/10.1016/j.matlet.2018.01.131

[S7] D. Xu, P. Wang, R. Yang, Nitrogen-doped carbon decorated $NaTi_2(PO_4)_3$ composite as an anode for sodium-ion batteries with outstanding electrochemical performance. Ceram. Int. **44**, 7159-7164 (2018). https://doi.org/10.1016/j.ceramint.2018.01.160

[S8] Z. Huang, L. Liu, L. Yi, W. Xiao, M. Li et al., Facile solvothermal synthesis of $NaTi_2(PO_4)_3$/C porous plates as electrode materials for high-performance sodium ion batteries. J. Power Sources **325**, 474-481(2016). https://doi.org/10.1016/j.jpowsour.2016.06.066

[S9] Y. Jiang, J. Shi, M. Wang, L. Zeng, L. Gu, Y. Yu, Highly reversible and ultrafast sodium storage in $NaTi_2(PO_4)_3$ nanoparticles embedded in nanocarbon networks. ACS Appl. Mater. Interfaces **8**, 689-695 (2016). https://doi.org/10.1021/acsami.5b09811

[S10] Y. Niu, M. Xu, C. Guo, C.M. Li, Pyro-synthesis of a nanostructured $NaTi_2(PO_4)_3$/C with a novel lower voltage plateau for rechargeable sodium-ion batteries. J. Colloid Interf. Sci. **474**, 88-92(2016). https://doi.org/10.1016/j.jcis.2016.04.021

[S11] D. Wang, Q. Liu, C. Chen, M. Li, X. Meng et al., NASICON-structured $NaTi_2(PO_4)_3$@C nanocomposite as the low operation-voltage anode material for high-performance sodium ion batteries. ACS Appl. Mater. Interfaces **8**, 2238-2246(2016). https://doi.org/10.1021/acsami.5b11003

[S12] J. Peng, J. Wang, H. Yi, W. Hu, Y. Yu et al., A dual-insertion type sodium-ion full cell based on high-quality ternary-metal prussian blue analogs. Adv. Energy Mater. **8**, 1702856 (2018). https://doi.org/10.1002/aenm.201702856

[S13] L. Fu, X. Xue, Y. Tang, D. Sun, H. Xie, H. Wang, Size controlling and surface engineering enable $NaTi_2(PO_4)_3$/C outstanding sodium storage properties. Electrochim. Acta **289**, 21-28(2018). https://doi.org/10.1016/j.electacta.2018.09.024

[S14] Y. Niu, M. Xu, Y. Zhang, J. Han, Y. Wang, C.M. Li, Detailed investigation of a $NaTi_2(PO_4)_3$ anode prepared by pyro-synthesis for Na-ion batteries. RSC Adv. **6**, 45605-45611(2016). https://doi.org/10.1039/C6RA06533C

[S15] H. Liu, H. Zhang, C. Su, X. Li, Y. Guo, Three-dimensional $NaTi_2(PO_4)_3$@C microsphere as a high-performance anode material for advanced sodium-ion batteries. Solid State Ionics **322**, 79-84 (2018). https://doi.org/10.1016/j.ssi.2018.05.005

[S16] L. Zhang, X. Wang, W. Deng, X. Zang, C. Liu et al., An open holey structure enhanced rate capability in a $NaTi_2(PO_4)_3$/C nanocomposite and provided ultralong-life sodium-ion storage. Nanoscale **10**, 958-963 (2018). https://doi.org/10.1039/C7NR07000D

[S17] Q. Hu, M. Yu, J. Liao, Z. Wen, C. Chen, Porous carbon-coated $NaTi_2(PO_4)_3$ with superior rate and low-temperature properties. J. Mater. Chem. A **6**, 2365-2370 (2018). https://doi.org/10.1039/C7TA10207K

[S18] M. Li, L. Liu, P. Wang, J. Li, Q. Leng, G. Cao, Highly reversible sodium-ion storage in $NaTi_2(PO_4)_3$/C composite nanofibers. Electrochim. Acta **252**, 523-531 (2017). https://doi.org/10.1016/j.electacta.2017.09.020

[S19] P. Wei, Y. Liu, Z. Wang, Y. Huang, Y. Jin et al., Porous $NaTi_2(PO_4)_3$/C hierarchical nanofibers for ultrafast electrochemical energy storage. ACS Appl. Mater. Interfaces **10**, 27039-27046 (2018). https://doi.org/10.1021/acsami.8b08415

[S20] S. Yu, Y. Wan, C. Shang, Z. Wang, L. Zhou, J. Zou, H. Cheng, Z. Lu, Ultrafine $NaTi_2(PO_4)_3$ nanoparticles encapsulated in N-CNFs as ultra-stable electrode for sodium storage. Front. Chem. **6**, 270 (2018). https://doi.org/10.3389/fchem.2018.00270

[S21] L. Wang, B. Wang, G. Liu, T. Liu, T. Gao, D. Wang, Carbon nanotube decorated $NaTi_2(PO_4)_3$/C nanocomposite for a high-rate and low-temperature sodium-ion battery anode. RSC Adv. **6**, 70277-70283 (2016). https://doi.org/10.1039/C6RA11042H

[S22] G.B. Xu, L.W. Yang, X.L. Wei, J.W. Ding, J.X. Zhong, P.K. Chu, Hierarchical porous nanocomposite architectures from multi-wall carbon nanotube threaded mesoporous $NaTi_2(PO_4)_3$ nanocrystals for high-performance sodium electrodes. J. Power Sources **327**, 580-590 (2016). https://doi.org/10.1016/j.jpowsour.2016.07.089

[S23] M. Bian, L. Tian, Design and synthesis of three-dimensional $NaTi_2(PO_4)_3$@CNT microspheres as advanced anode materials for rechargeable sodium-ion batteries. Ceram. Int. **43**, 9543-9546 (2017). https://doi.org/10.1016/j.ceramint.2017.04.029

[S24] Z. Wang, J. Liang, K. Fan, X. Liu, C. Wang, J. Ma, Porous $NaTi_2(PO_4)_3$ nanocubes anchored on porous carbon nanosheets for high performance sodium-ion batteries. Front. Chem. **6**, (2018). https://doi.org/10.3389/fchem.2018.00396

[S25] G. Pang, P. Nie, C. Yuan, L. Shen, X. Zhang, H. Li, C. Zhang, Mesoporous $NaTi_2(PO_4)_3$/CMK-3 nanohybrid as anode for long-life Na-ion batteries. J. Mater. Chem. A **2**, 20659-20666 (2014). https://doi.org/10.1039/C4TA04732J

[S26] H. Liu, Y. Liu, 1D mesoporous $NaTi_2(PO_4)_3$/carbon nanofiber: The promising anode material for sodium-ion batteries. Ceram. Int. **44**, 5813-5816 (2018). https://doi.org/10.1016/j.ceramint.2017.12.147

[S27] J. Liang, K. Fan, Z. Wei, X. Gao, W. Song, J. Ma, Porous $NaTi_2(PO_4)_3$@C nanocubes as improved anode for sodium-ion batteries. Mater. Res. Bull. **99**, 343-348 (2018). https://doi.org/10.1016/j.materresbull.2017.11.030

[S28] Y. Jiang, L. Zeng, J. Wang, W. Li, F. Pan, Y. Yu, A carbon coated NASICON structure material embedded in porous carbon enabling superior sodium storage performance: $NaTi_2(PO_4)_3$ as an example. Nanoscale **7**, 14723-14729(2015). https://doi.org/10.1039/C5NR03978A

[S29] J. Yang, H. Wang, P. Hu, J. Qi, L. Guo, L. Wang, A high-rate and ultralong-life sodium-ion battery based on $NaTi_2(PO_4)_3$ nanocubes with synergistic coating of carbon and rutile TiO_2. Small **11**, 3744-3749 (2015). https://doi.org/10.1002/smll.201500144

[S30] C. Xu, Y. Xu, C. Tang, Q. Wei, J. Meng, L. Huang, L. Zhou, G. Zhang, L. He, L. Mai, Carbon-coated hierarchical $NaTi_2(PO_4)_3$ mesoporous microflowers with superior sodium storage performance. Nano Energy **28**, 224-231(2016). https://doi.org/10.1016/j.nanoen.2016.08.026

[S31] X. Li, X. Zhu, J. Liang, Z. Hou, Y. Wang, N. Lin, Y. Zhu, Y. Qian, Graphene-supported $NaTi_2(PO_4)_3$ as a high rate anode material for aqueous sodium ion batteries. J. Electrochem. Soc. **161**, A1181-A1187 (2014). https://doi.org/10.1149/2.0081409jes

[S32] J.S. Ko, C.S. Choi, B. Dunn, J.W. Long, Electrochemical characterization of Na-ion charge-storage properties for nanostructured $NaTi_2(PO_4)_3$ as a function of crystalline order. J. Electrochem. Soc. **164**, A2124-A2130(2017). https://doi.org/10.1149/2.1391709jes

[S33] M. Sun, X. Han, S. Chen, $NaTi_2(PO_4)_3$@C nanoparticles embedded in 2D sulfur-doped graphene sheets as high-performance anode materials for sodium energy storage. Electrochim. Acta **289**,131-138 (2018). https://doi.org/10.1016/j.electacta.2018.08.061

[S34] H.-K. Roh, H.-K. Kim, M.-S. Kim, D.-H. Kim, K.Y. Chung, K.C. Roh, K.-B. Kim, In situ synthesis of chemically bonded $NaTi_2(PO_4)_3$/rGO 2D nanocomposite for high-rate sodium-ion batteries. Nano Res. **9**, 1844-1855(2016). https://doi.org/10.1007/s12274-016-1077-y

[S35] J. Song, S. Park, J. Gim, V. Mathew, S. Kim, J. Jo, S. Kim, J. Kim, High rate performance of a $NaTi_2(PO_4)_3$/rGO composite electrode via pyro synthesis for sodium ion batteries. J. Mater. Chem. A **4**,7815-7822(2016). https://doi.org/10.1039/C6TA02720B

[S36] Y. Hu, X. Ma, P. Guo, F. Jaeger, Z. Wang, Design of $NaTi_2(PO_4)_3$ nanocrystals embedded in N-doped graphene sheets for sodium-ion battery anode with superior electrochemical performance. Ceram. Int. **43**, 12338-12342(2017). https://doi.org/10.1016/j.ceramint.2017.06.098

[S37] S. Ye, Z. Li, T. Song, D. Cheng, Q. Xu, H. Liu, Y. Wang, Self-generated hollow $NaTi_2(PO_4)_3$ nanocubes decorated with graphene as a large capacity and long lifetime anode for sodium-ion batteries. RSC Adv. **7**, 56743-56751(2017). https://doi.org/10.1039/C7RA12291H

[S38] X. Jiang, Z. Zeng, L. Xiao, X. Ai, H. Yang, Y. Cao, An all-phosphate and zero-strain sodium-ion battery based on $Na_3V_2(PO_4)_3$ Cathode, $NaTi_2(PO_4)_3$ anode, and trimethyl phosphate electrolyte with intrinsic safety and long lifespan. ACS Appl. Mater. Interfaces **9**, 43733-43738 (2017). https://doi.org/10.1021/acsami.7b14946

[S39] Y. Zuo, L. Chen, Z. Zuo, Y. Huang, X. Liu, Rational construction of $NaTi_2(PO_4)_3$@C nanocrystals embedded in graphene sheets as anode materials for Na-ion batteries. Ceram. Int. **43**,12915-12919(2017). https://doi.org/10.1016/j.ceramint.2017.06.189

[S40] Q. Yang, T. Jiao, M. Li, Y. Li, L. Ma et al., In situ formation of $NaTi_2(PO_4)_3$ cubes on Ti3C2 MXene for dual-mode sodium storage. J. Mater. Chem. A **6**, 18525-18532(2018). https://doi.org/10.1039/C8TA06995F

[S41] C. Yang, X. Sun, Y.R. Zhang, Y. Liu, Q.A. Zhang, C.Z. Yuan, Facile synthesis of hierarchical $NaTi_2(PO_4)_3/Ti_3C_2$ nanocomposites with superior sodium storage performance. Mater. Lett. **236**, 408-411(2019). https://doi.org/10.1016/j.matlet.2018.10.147

[S42] D. Guo, J. Qin, C. Zhang, M. Cao, Constructing flexible and binder-free $NaTi_2(PO_4)_3$ film electrode with a sandwich structure by a two-step graphene hybridizing strategy as an ultrastable anode for long-life sodium-ion batteries. Cryst. Growth Des. **18**, 3291-3301(2018). https://doi.org/10.1021/acs.cgd.7b01549

[S43] L. Xu, G. Xu, Z. Chen, X. Wei, J. Cao, L. Yang, 3D nanocomposite archiecture constructed by reduced graphene oxide, thermally-treated protein and mesoporous $NaTi_2(PO_4)_3$ nanocrystals as free-standing electrodes for advanced sodium ion battery. J. Mater. Sci. **29**, 9258-9267(2018). https://doi.org/10.1007/s10854-018-8955-x

[S44] H. Zhang, B. Qin, D. Buchholz, S. Passerini, High-efficiency sodium-ion battery based on NASICON electrodes with high power and long lifespan. ACS Appl. Energy Mater. (2018). https://doi.org/10.1021/acsaem.8b01390

[S45] Z. Zhou, N. Li, C. Zhang, X. Chen, F. Xu, C. Peng, Preparation of carbon layer and carbon nanotube co-decorated $NaTi_2(PO_4)_3$ anode and its application in sodium-ion batteries. Solid State Ionics **324**, 87-91 (2018). https://doi.org/10.1016/j.ssi.2018.06.011

[S46] G. Xu, L. Yang, Z. Li, X. Wei, P.K. Chu, Protein-assisted assembly of mesoporous nanocrystals and carbon nanotubes for self-supporting high-performance sodium electrodes. J. Mater. Chem. A **5**, 2749-2758 (2017). https://doi.org/10.1039/C6TA09673E

[S47] G. Xu, Z. Li, X. Wei, L. Yang, P.K. Chu, Monolithic hierarchical carbon assemblies embedded with mesoporous $NaTi_2(PO_4)_3$ nanocrystals for flexible high-performance sodium anodes. Electrochim. Acta **254**, 328-336 (2017). https://doi.org/10.1016/j.electacta.2017.09.121

[S48] K. Zhao, Y. Liu, S. Zhang, S. He, N. Zhang, J. Yang, Z. Zhan, A room temperature solid-state rechargeable sodium ion cell based on a ceramic Na-beta''-Al_2O_3 electrolyte and $NaTi_2(PO_4)_3$ cathode. Electrochem. Commun. **69**, 59-63 (2016). https://doi.org/10.1016/j.elecom.2016.06.003

[S49] W. Zhou, Y. Li, S. Xin, J.B. Goodenough, Rechargeable sodium all-solid-state battery. ACS Central Sci. **3**, 52-57 (2017). https://doi.org/10.1021/acscentsci.6b00321

[S50] S.I. Park, I. Gocheva, S. Okada, J.-i. Yamaki, Electrochemical properties of $NaTi_2(PO_4)_3$ anode for rechargeable aqueous sodium-ion batteries. J. Electrochem. Soc. **158**, A1067-A1070 (2011). https://doi.org/10.1149/1.3611434

[S51] Y. He, Y. Hua, Y. Wu, C. Chi, Y. Shi, C. Ai, $NaTi_2(PO_4)_3$/carbon and $NaTi_2(PO_4)_3$/graphite composites as anode materials for aqueous rechargeable na-ion batteries. Electrochemistry **84**, 705-708 (2016). https://doi.org/10.5796/electrochemistry.84.705

[S52] L. Chen, J. Liu, Z. Guo, Y. Wang, C. Wang, Y. Xia, Electrochemical profile of $LiTi_2(PO_4)_3$ and $NaTi_2(PO_4)_3$ in lithium, sodium or mixed ion aqueous solutions. J. Electrochem. Soc. **163**, A904-A910 (2016). https://doi.org/10.1149/2.0531606jes

[S53] T.-F. Hung, W.-H. Lan, Y.-W. Yeh, W.-S. Chang, C.-C. Yang, J.-C. Lin, Hydrothermal synthesis of sodium titanium phosphate nanoparticles as efficient anode materials for aqueous sodium-ion batteries. ACS Sustain. Chem. Eng. **4**, 7074-7079 (2016). https://doi.org/10.1021/acssuschemeng.6b01962

[S54] A.I. Mohamed, N.J. Sansone, B. Kuei, N.R. Washburn, J.E. Whitacre, Using polypyrrole coating to improve cycling stability of $NaTi_2(PO_4)_3$ as an aqueous Na-ion anode. J. Electrochem. Soc. **162**, A2201-A2207 (2015). https://doi.org/10.1149/2.0961510jes

[S55] X. Yao, Y. Luo, Y. Li, W. Li, M. Fang, M. Shui, J. Shu, Y. Ren, The investigation of $NaTi_2(PO_4)_3$@C/Ag as a high-performance anode material for aqueous rechargeable sodium-ion batteries. Mater. Res. Bull. **104**, 194-201(2018). https://doi.org/10.1016/j.materresbull.2018.03.035

[S56] X. Cao, Y. Yang, Facile synthesis of $NaTi_2(PO_4)_3$-carbon composite through solid state method and its application in aqueous sodium ion battery. Mater. Lett. **231**, 183-186 (2018). https://doi.org/10.1016/j.matlet.2018.08.020

[S57] W. Wu, J. Yan, A. Wise, A. Rutt, J.F. Whitacre, Using intimate carbon to enhance the performance of $NaTi_2(PO_4)_3$ anode materials: carbon nanotubes vs graphite. J. Electrochem. Soc. **161**, A561-A567 (2014). https://doi.org/10.1149/2.059404jes

[S58] W. Wu, A. Mohamed, J.F. Whitacre, Microwave synthesized $NaTi_2(PO_4)_3$ as an aqueous sodium-ion negative electrode. J. Electrochem. Soc. **160**, A497-A504 (2013). https://doi.org/10.1149/2.054303jes

[S59] B. Zhao, Q. Wang, S. Zhang, C. Deng, Self-assembled wafer-like porous $NaTi_2(PO_4)_3$ decorated with hierarchical carbon as a high-rate anode for aqueous rechargeable sodium batteries. J. Mater. Chem. A **3**, 12089-12096 (2015). https://doi.org/10.1039/C5TA02568K

[S60] B. Zhao, B. Lin, S. Zhang, C. Deng, A frogspawn-inspired hierarchical porous $NaTi_2(PO_4)_3$-C array for high-rate and long-life aqueous rechargeable sodium batteries. Nanoscale **7**, 18552-18560 (2015). https://doi.org/10.1039/C5NR06505D

[S61] G. Pang, C. Yuan, P. Nie, B. Ding, J. Zhu, X. Zhang, Synthesis of NASICON-type structured $NaTi_2(PO_4)_3$-graphene nanocomposite as an anode for aqueous rechargeable Na-ion batteries. Nanoscale **6**, 6328-6334 (2014). https://doi.org/10.1039/C3NR06730K

[S62] Z. Li, D. Young, K. Xiang, W.C. Carter, Y.-M. Chiang, Towards high power high energy aqueous sodium-ion batteries: The $NaTi_2(PO_4)_3/Na_{0.44}MnO_2$ system. Adv. Energy Mater. **3**, 290-294 (2013). https://doi.org/10.1002/aenm.201200598

[S63] Q. Zhang, C. Liao, T. Zhai, H. Li, A high rate 1.2V aqueous sodium-ion battery based on all NASICON structured $NaTi_2(PO_4)_3$ and $Na_3V_2(PO_4)_3$. Electrochim. Acta **196**, 470-478 (2016). https://doi.org/10.1016/j.electacta.2016.03.007

[S64] Z. Liu, Y. An, G. Pang, S. Dong, C. Xu, C. Mi, X. Zhang, TiN modified $NaTi_2(PO_4)_3$ as an anode material for aqueous sodium ion batteries. Chem. Eng. J. **353**, 814-823(2018). https://doi.org/10.1016/j.cej.2018.07.159

[S65] X. Wu, Y. Cao, X. Ai, J. Qian, H. Yang, A low-cost and environmentally benign aqueous rechargeable sodium-ion battery based on $NaTi_2(PO_4)_3$-$Na_2NiFe(CN)_6$ intercalation chemistry. Electrochem. Commun. **31**, 145-148(2013). https://doi.org/10.1016/j.elecom.2013.03.013

[S66] Z. Hou, X. Li, J. Liang, Y. Zhu, Y. Qian, An aqueous rechargeable sodium ion battery based on a $NaMnO_2$- $NaTi_2(PO_4)_3$ hybrid system for stationary energy storage. J. Mater. Chem. A **3**, 1400-1404 (2015). https://doi.org/10.1039/C4TA06018K

[S67] X.-y. Wu, M.-y. Sun, Y.-f. Shen, J.-f. Qian, Y.-l. Cao, X.-p. Ai, H.-x. Yang, Energetic aqueous rechargeable sodium-ion battery based on $Na_2CuFe(CN)_6$- $NaTi_2(PO_4)_3$ intercalation chemistry. Chemsuschem **7**, 407-411(2014). https://doi.org/10.1002/cssc.201301036

[S68] G. Pang, P. Nie, C. Yuan, L. Shen, X. Zhang, J. Zhu, B. Ding, Enhanced performance of aqueous sodium-ion batteries using electrodes based on the $NaTi_2(PO_4)_3$/MWNTs-$Na_{0.44}MnO_2$ system. Energy Technol. **2**, 705-712 (2014). https://doi.org/10.1002/ente.201402045
